# Identification of Intermediate in Evolutionary Model of Enterohemorrhagic *Escherichia coli* O157

**DOI:** 10.3201/eid1804.111414

**Published:** 2012-04

**Authors:** Christian Jenke, Shana R. Leopold, Thomas Weniger, Jörg Rothgänger, Dag Harmsen, Helge Karch, Alexander Mellmann

**Affiliations:** Institute for Hygiene and National Consulting Laboratory on Hemolytic Uremic Syndrome, Münster, Germany (C. Jenke, S.R. Leopold, H. Karch, A. Mellmann);; University Hospital Münster Periodontology, Münster (T. Weniger, D. Harmsen);; Ridom GmbH, Münster (J. Rothgänger)

**Keywords:** EHEC O157, SNP typing, single nucleotide polymorphism, sorbitol fermentation, phylogeny, evolution, enterohemorrhagic Escherichia coli, Escherichia coli, E. coli, bacteria, deer, human, pathogen, *Suggested citation for this article*: Jenke C, Leopold SR, Weniger T, Rothgänger J, Harmsen D, Karch H, et al. Identification of intermediate in evolutionary model of enterohemorrhagic *Escherichia coli* O157. Emerg Infect Dis [serial on the internet]. 2012 Apr [*date cited*]. http://dx.doi.org/10.3201/eid1804.111414

## Abstract

Highly pathogenic enterohemorrhagic *Escherichia coli* (EHEC) O157 cause a spectrum of clinical signs that include diarrhea, bloody diarrhea, and hemolytic uremic syndrome. The current evolutionary model of EHEC O157:H7/H^–^ consists of a stepwise evolution scenario proceeding from O55:H7 to a node (hypothetical intermediate) that then branches into sorbitol-fermenting (SF) O157:H^–^ and non-SF (NSF) O157:H7. To identify this hypothetical intermediate, we performed single nucleotide polymorphism analysis by sequencing of 92 randomly distributed backbone genomic regions of 40 O157:H7/H^–^ isolates. Overall, 111 single nucleotide polymorphisms were identified in 75/92 partial open reading frames after sequencing 51,041 nt/strain. The EHEC O157:H7 strain LSU-61 from deer occupied an intermediate position between O55:H7 and both O157 branches (SF and NSF O157), complementing the stepwise evolutionary model of EHEC O157:H7/H^–^. The animal origin of this intermediate emphasizes the value of nonhuman reservoirs in the clarification of the evolution of human pathogens.

## Abstract 2

Single-nucleotide polymorphism typing found missing link between human strains in strain from deer.

Enterohemorrhagic *Escherichia coli* (EHEC) belongs to the Shiga toxin–producing *E. coli* group and causes clinical signs ranging from watery to bloody diarrhea for most symptomatically infected patients (*1**,**2*). EHEC serotypes O157:H7 and O157:H^–^ (nonmotile) are the most frequently isolated from patients with severe EHEC-associated diseases, such as bloody diarrhea and hemolytic uremic syndrome. Infections caused by EHEC O157:H7/H^–^ are major public health threats and require considerable resources for control and prevention (*1**,**3*). Sorbitol-fermenting (SF) EHEC O157:H^–^, initially found in Germany and later in other countries such as Scotland, Finland, and Australia, are increasingly associated with severe disease (*4*). These strains can ferment sorbitol after overnight incubation on sorbitol MacConkey agar, unlike non-SF (NSF) EHEC O157:H7. Today, SF EHEC O157:H^–^ strains cause ≈20% of all hemolytic uremic syndrome cases in Germany (*4**–**8*). Classic NSF EHEC O157:H7 are of animal origin and have caused multiple outbreaks through contaminated food (*4*), but SF EHEC O157:H^–^ are almost exclusively isolated from humans, which suggests that humans are the main reservoir (*5*).

On the basis of multilocus enzyme electrophoresis and multilocus sequence typing (MLST) data (*9**,**10*), the evolutionary model of EHEC O157 suggests that EHEC O157 emerged from *E. coli* O55:H7 by loss and acquisition of virulence and phenotypic traits (*10*). To further explain the evolution from O55:H7, a hypothetical intermediate and putatively extinct clone (missing link) SF O157:H7 emerging from O55:H7 was introduced; theoretically, it is from this intermediate that the 2 branches (NSF O157:H7 and SF O157:H^–^) diverged (*9**,**10*).

Shaikh and Tarr subdivided NSF O157:H7 into 3 clusters (*11*); in their analysis, the SF O157:H^–^ branch remained evolutionary conserved and clearly separated from NSF O157:H7, with additional data suggesting a hypothetical intermediate. Recent studies based on whole core genome single-nucleotide polymorphisms (SNPs) enabled precise reconstruction of this model (*12*). The *E. coli* O157:H^–^ strain LSU-61, which was isolated from a deer (*10**,**13*), had been previously discussed by Feng et al. as a potential intermediate, but that hypothesis was rejected because the strain lacked a gene encoding Shiga toxin (*stx*) and had a distinct MLST sequence type (*10*). We used an SNP-based approach to examine isolates from different sources of EHEC O157:H7/H^–^ to further elucidate the evolutionary model of emergence of this pathogen, paying particular attention to identifying the “missing link” hypothetical intermediate.

## Materials and Methods

### Bacterial Strains Analyzed

Of the 50 EHEC strains examined (Table), 48 were serotype O157:H7/H^–^ and 2 were O55:H7. Core or complete genome sequences were available for 8 O157 and 2 O55:H7 strains; these sequences served as a framework of the evolutionary model of EHEC O157. The remaining 40 strains consisted of 13 O157:H7/H^–^ strains that represented different clusters according to previous multilocus variable-number tandem-repeat analysis (*19*); 26 O157:H7/H^–^ strains isolated during 1987–2010 that were randomly chosen from our strain collection; and strain LSU-61, which was considered to be an intermediate (*10*).

**Table:** Fifty strains used for SNP typing of enterohemorrhagic *Escherichia coli* O157*

Strain ID	Year of isolation	Illness	SF status and serotype	Subgroup/cluster†	Reference and/or GenBank accession no.
TB182A‡	1991	D	SF O55:H7	**A**	(*12*)
CB9615‡	2003	D	SF O55:H7	A	NC_013941
493/89‡	1989	HUS	SF O157:H^–^	**B**	(*16*)
87–14‡	1987	HUS	NSF O157:H7	**C1**	(*12*)
EC4115‡	2006	BD	NSF O157:H7	C1	NC_011353
TW14359‡	2006	BD	NSF O157:H7	**C1**	NC_013008 (*12**,**17*)
TW14588‡	2006	BD	NSF O157:H7	C3	NZ_ABKY00000000.2
86–24‡	1986	HUS	NSF O157:H7	**C2**	(*12*)
Sakai‡	1996	D	NSF O157:H7	**C3**	NC_002695 (*18*)
EDL933‡	1983	NA	NSF O157:H7	**C3**	(*14*)
LSU-61	2001	NA	SF O157:H7	Unknown intermediate	(*10**,**13*)
SNPO157_01	1987	HUS	NSF O157:H7	C1	This study
SNPO157_02	1988	D	NSF O157:H7	C1	This study
SNPO157_03	1988	HUS	SF O157:H^–^	B	This study
SNPO157_04	1990	HUS	NSF O157:H7	C1	This study
SNPO157_05	1991	HUS	NSF O157:H^–^	C3	This study
SNPO157_06	1992	HUS	NSF O157:H7	C3	This study
SNPO157_07	1993	HUS	NSF O157:H7	C1	This study
SNPO157_08	1995	HUS	NSF O157:H7	C1	This study
SNPO157_09	1995	HUS	SF O157:H^–^	B	This study
SNPO157_10	1996	HUS	NSF O157:H^–^	C1	This study
SNPO157_11	1996	HUS	SF O157:H^–^	B	This study
SNPO157_12	1996	HUS	SF O157:H^–^	B	This study
SNPO157_13	1996	HUS	NSF O157:H7	C1	This study
SNPO157_14	1997	HUS	NSF O157:H7	C1	This study
SNPO157_15	1997	HUS	NSF O157:H7	C1	This study
SNPO157_16	1998	HUS	NSF O157:H7	C1	This study
SNPO157_17	1999	HUS	NSF O157:H7	C1	This study
SNPO157_18	1999	HUS	NSF O157:H7	C3	This study
SNPO157_19	2000	HUS	NSF O157:H7	C1	This study
SNPO157_20	2000	D	NSF O157:H7	C3	This study
SNPO157_21	2001	HUS	SF O157:H^–^	B	This study
SNPO157_22	2001	HUS	NSF O157:H^–^	C3	This study
SNPO157_23	2002	D	NSF O157:H7	C3	This study
SNPO157_24	2002	A	NSF O157:H7	C1	This study
SNPO157_25	2003	HUS	NSF O157:H7	C3	This study
SNPO157_26	2004	HUS	NSF O157:H7	C1	This study
SNPO157_27	2005	HUS	NSF O157:H7	C3	This study
SNPO157_28	2005	BD	NSF O157:H7	C3	This study
SNPO157_29	2005	HUS	NSF O157:H7	C1	This study
SNPO157_30	2006	HUS	NSF O157:H7	C3	This study
SNPO157_31	2007	HUS	NSF O157:H7	C3	This study
SNPO157_32	2007	HUS	NSF O157:H7	C3	This study
SNPO157_33	2008	HUS	NSF O157:H7	C3	This study
SNPO157_34	2008	D	SF O157:H^–^	B	This study
SNPO157_35	2008	HUS	SF O157:H^–^	B	This study
SNPO157_36	2009	D	NSF O157:H7	§	This study
SNPO157_37	2009	HUS	SF O157:H^–^	B	This study
SNPO157_38	2010	HUS	NSF O157:H7	C3	This study
SNPO157_39	2010	HUS	SF O157:H^–^	B	This study

*All strains were isolated from humans except strain LSU-61, which was isolated from a deer (*10*), and EDL933, which was isolated from food (*14*). Strains isolated from humans were categorized into 3 subgroups (*11**,**12*); subgroup A represents isolates of serotype O55:H7, subgroup B SF O157:H– isolates, and subgroup C NSF O157:H7. Subgroup C is subdivided into clusters 1–3. SNP, single-nucleotide polymorphism; ID, identification; SF, sorbitol fermenting; D, diarrhea; HUS, hemolytic uremic syndrome; NSF, non-SF; BD, bloody diarrhea; NA, not applicable; A, asymptomatic. †Subgroup and, if applicable, cluster designation based on 4 SNP loci (Sakai genome positions 337933, 1460599, 2370797, and 5404166) and the occupancy of potential *stx* integration sites in accordance with (*11**,**12**,**15*). **Boldface** indicates cluster designation of prototype strains. ‡Strains were analyzed in silico. §SNP pattern for NSF O157:H7 grouping resulted in an unknown combination.

### Identification of EHEC O157 Strains

All 39 EHEC O157 isolates from our laboratory were isolated from stool samples as described (*20**,**21*). Isolates were confirmed to be *E. coli* by the API 20 Etest (bioMérieux, Marcy l’Etoile, France) and serotyped by using antiserum against *E. coli* O antigens 1–181 and H antigens 1–56 (*22*). Subtyping of *fliC* genes in nonmotile isolates by using *Hha*I restriction fragment-length polymorphism of amplicons obtained with primers FSa1 and rFSa1 (*23**,**24*) confirmed the presence of *fliC*_H7_ in all isolates. All strains were frozen at −70°C until further use.

### Isolation of DNA

A single colony from a fresh overnight culture on Columbia blood agar (Heipha, Eppelheim, Germany) was inoculated into a liquid culture of nutrient broth medium (Heipha) and incubated overnight at 37°C. The liquid culture was used to prepare DNA as described (*25*), except that phenol extraction was omitted and the corresponding supernatants were directly precipitated with isopropanol.

### Cluster Classification of O157:H7 Strains

Previously determined SNP patterns T/G/T/A or G/T/C/C at Sakai genome positions 337,933 (ECs0320, putative receptor), 1,460,599 (ECs1414, curli production assembly/transport component), 2,370,797 (ECs2397, transport system permease protein), and 5,404,166 (ECs5279, *fimH–*locus) have been shown to be cluster specific (*12*). On this basis, we used Sanger sequencing to group strains into cluster 3 or cluster 1 of subgroup C. Because the prototype strain of cluster 2 shared the SNP pattern with cluster 3, strains of cluster 2 were differentiated by using the published cluster differentiation scheme based on the occupancy of *stx* integration sites and the *stx* genotype (*11**,**15*). SNP pattern T/G/T/C was declared as unknown.

### MLST and Sequencing of EHEC O157 Core Genomic Loci

As a first classification, we used MLST to determine the sequence type (ST)for all prototype strains of each subgroup and cluster by sequencing internal fragments of 7 housekeeping genes (*adk*, *fumC*, *gyrB*, *icd*, *mdh*, *purA*, and *recA*) (*26*). Alleles, STs, and clonal complexes were assigned in accordance with the *E. coli* MLST website (http://mlst.ucc.ie/mlst/dbs/Ecoli).

In addition to 10 SNP localizations that were known to differentiate subgroups and clusters (*12*), we randomly selected 82 additional backbone genomic regions for more in-depth SNP analysis. Using the Primer3 algorithm (http://frodo.wi.mit.edu/primer3), we developed 93 primer pairs that generated PCR products of backbone genomic regions ranging from 600 to 700 bp (Technical Appendix Table 1); for open reading frame (ORF) ECs3076, 2 separate primer pairs were designed to cover 2 described SNP localizations (*12*). EHEC O157:H7 strain Sakai served as a reference (GenBank accession no. NC_002655).

PCR was performed in a 14-µL reaction mixture containing 7 µL REDTaq (Sigma Aldrich, St. Louis, MO, USA), ≈6 ng DNA, and 1.5 µL each forward and reverse primer, with a final concentration of 10 µmol/L. The cycling reaction conditions were initial denaturation (2 min at 94°C), 35 cycles of denaturation (45 s at 94°C), annealing (60 s at 60°C), and extension (90 s at 72°C), followed by a final extension (10 min at 72°C). PCR products were purified by using the exonuclease I (New England Biolabs GmbH, Frankfurt-Hoechst, Germany) and shrimp alkaline phosphatase (USB Amersham, Freiburg, Germany) according to methods modified from (*27*). In brief, 7 µL of the PCR product was incubated simultaneously with 1.5 U of each enzyme at 37°C for 45 min, followed by enzyme heat inactivation at 80°C.

For sequencing of both strands, 2 µL of the purified amplicons was mixed with 0.5 µL premix from the ABI Prism BigDye Terminator v3.1 Ready Reaction Cycle Sequencing Kit (Applied Biosystems, Foster City, CA, USA) plus 1.8 µL Tris-HCl-MgCl_2_ buffer (400 mmol/L Tris-HCl, 10 mmol/L MgCl_2_; pH 9) and 2 µL (10 µmol/L) from the sequencing primer (forward or reverse primer, in a total volume of 10 µL. The cycling reaction conditions were 25 cycles of denaturation (10 s at 96°C) and combined annealing and extension (4 min at 60°C). Finally, the sequencing reaction products were purified by using an alcohol precipitation method as recommended by the manufacturer and loaded onto a 3130xl Genetic Analyzer (Applied Biosystems) for capillary sequencing.

### Genotypic Characterization of LSU-61

To further evaluate the genotype of LSU-61 and its potential role in the evolutionary model of EHEC O157, we investigated known *stx-*phage integration sites. We used the draft genome sequence of the O157 strain LSU-61 (GenBank accession no. AEUC00000000) (*28*). *yehV*, a known integration site of *stx1*, was screened in silico by using primer pair A/B from (*29*). For analysis of the *wrbA* locus, a site of integration of the *stx2* bacteriophage, we used primer pair C/D from (*29*). The 2 other currently known potential integration sites of *stx2*, *yecE* and *sbcB*, were screened by using primer pairs EC10/EC11, yecD-fwd/yecN-rev, and sbcB1/sbcB2 (*30*).

### Data Analysis

Sequence trace files were analyzed and stored by using SeqSphere software version 0.9 beta (Ridom GmbH, Münster, Germany); a minimum-spanning tree was constructed with the integrated minimum-spanning tree algorithm. Gene functions were categorized by using the Pathosystems Resource Integration Center database (www.patricbrc.org/portal/portal/patric/Home) and corresponding Kyoto Encyclopedia of Genes and Genomes (KEGG; www.genome.jp/kegg) assignments. Overall, genes were grouped into 3 functional categories: metabolism/housekeeping, putative metabolism/housekeeping, and hypothetical protein. If no KEGG phenotype assignment was found, a putative metabolism/housekeeping function was predicted on the basis of BLAST (http://blast.ncbi.mlm.nih.gov/Blast.cgi) results.

## Results

Of the 48 EHEC O157 strains studied, 10 were SF serotype O157:H^–^. Subgrouping and cluster designation of the NSF O157 strains resulted in 18 cluster 1 strains, 1 cluster 2 strain, and 17 cluster 3 strains. For 2 strains, LSU-61 and SNPO157_36, no characteristic SNP pattern was determined (Table). Further characterization by MLST of prototype strains that defined subgroups and clusters resulted in identical STs for all SF and NSF O157 (ST11) and in closely related STs of the O55:H7 strains (ST335).

The 50 strains of serotypes O157:H7/H^–^ and O55:H7 were further characterized with respect to their SNP prevalence in the core genome. In total, 92 core genomic loci were analyzed, comprising 51,041 bp sequencing information (≈0.9% of the O157:H7 Sakai genome) (Table; Technical Appendix Table 2). Sequencing demonstrated 111 biallelic variants, an average of 1.2 variants per sequenced locus (Technical Appendix Table 3). Deletions or insertions were not detected.

Of the 111 SNPs, 53 (47.7%) were synonymous SNPs (sSNPs) and 58 (52.3%) were nonsynonymous SNPs (nsSNPs); 78 (70.3%) SNPs were transitions, and 33 (29.7%) were transversions. Concatenation of all loci resulted in an average of 1 SNP every 460 bp; sSNPs occurred every 963 bp, and nsSNPs every 880 bp. On the level of analyzed partial ORFs, 1 SNP was found in 45 partial ORFs, 2 SNPs in 25 partial ORFs, 3 SNPs in 4 partial ORFs, and 4 SNPs in 1 partial ORF.

To further elucidate the SNP distribution, we categorized the 92 loci into 3 functional groups. Most loci belonged to (putative) metabolism or housekeeping genes because these were chosen solely from backbone regions. If no KEGG assignment was possible, we estimated the function of the corresponding fragment on the basis of BLAST homologies. Defined annotation information regarding the function in metabolism or housekeeping was determined for 25 partial ORFs. A housekeeping/metabolism function was predicted for 58 loci. The remaining 9 loci were hypothetical proteins only (Technical Appendix).

On the basis of the 111 SNPs, the 50 strains were clustered into 27 SNP genotypes (Figure). The 111 SNPs were able to reconstruct the well-known evolutionary model (*9**–**12*) with the stepwise evolution from O55:H7 (subgroup A) to either SF O157:H^–^ (subgroup B) or NSF O157:H7/H^–^ (subgroup C, clusters 1–3). Strain LSU-61 interlinked all 3 subgroups, thereby substituting for the unknown intermediate (Figure). Moreover, the applied SNP scheme differentiated the O55:H7 strains and exhibited 5 genotypes within the 10 SF O157:H^–^ strains segregated from the SF O157:H7 strain LSU-61. The NSF O157:H7 subgroup C_1_ strains comprising 18 isolates were differentiated in 9 SNP genotypes, and the subgroup C_3_ strains (n = 17) exhibited 8 SNP genotypes.

**Figure:**
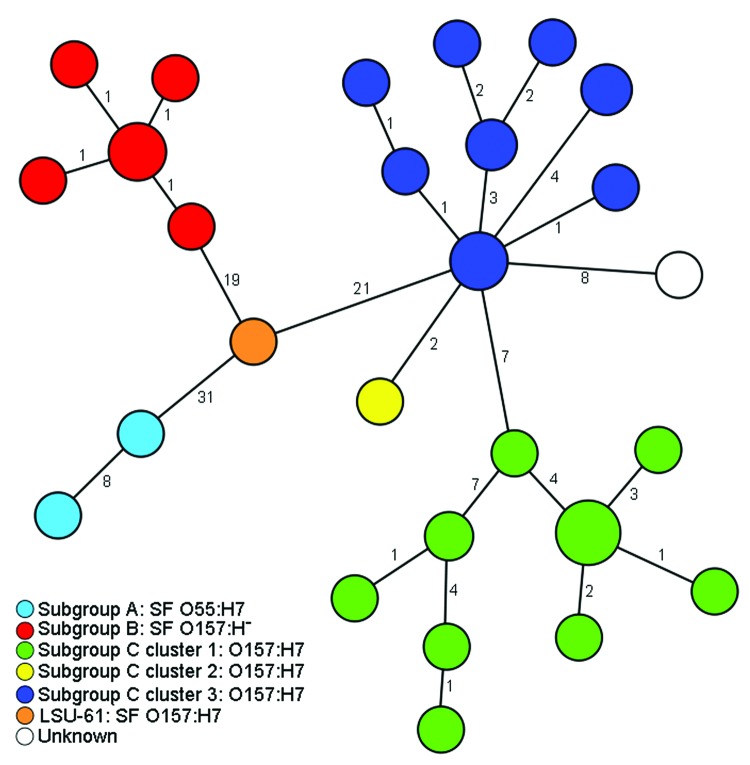
Minimum-spanning tree based on single-nucleotide polymorphism (SNP) genotypes illustrating the phylogeny of 50 enterohemorrhagic *Escherichia coli* O157:H7/H^–^ and O55:H7 isolates and the intermediate position of strain LSU-61 during the evolution of O157. Each node represents a unique SNP genotype. The size of each node is proportional to the number of isolates per SNP genotype based on sequence analysis of 51,041 bp comprising 92 partial open reading frames. Numbers on lines between nodes represent distances between the nodes, i.e., the number of SNPs. The node size is proportional to the number of strains sharing the same genotype. Strains are colored according to their classification into subgroups and clusters based on information from Saikh and Tarr (*11*) and Leopold et al. (*12*). Strain LSU-61 represents a potential intermediate interlinking all 3 subgroups. SF, sorbitol fermenting.

To further validate the role of *stx*-negative LSU-61 as a potential intermediate, we investigated each known potential *stx* insertion site in silico to determine the presence or absence of a Shiga toxin–carrying bacteriophage. We conducted BLAST searches within the recently published draft genome sequence of LSU-61 (*28*) by using published primers for the different insertion sites (*29*,*30*). All insertion sites for *stx1* (*yehV*) and *stx*2 (*wrbA*, *yecE*, *sbcB*) were intact.

To investigate the effect of selective pressure on some loci and potential selecting biases, we analyzed sSNP and nsSNP types separately. In each scenario, the phylogenetic reconstruction resulted in comparable branching, with distinct lineages for SF and NSF O157 and strain LSU-61 as an intermediate. Only the number of SNP genotypes differed slightly: 19 sSNP genotypes (13 NSF O157:H7, 3 SF O157:H^–^, 2 O55:H7, and LSU-61) based on the 53 sSNPs and 22 nsSNP genotypes (16 NSF O157:H7, 3 SF O157:H^–^, 2 O55:H7, and LSU-61) based on the 58 nsSNPs. This excludes strong selection bias of the different loci.

## Discussion

On the basis of SNP analysis of 92 chromosomal backbone regions of EHEC O157, we identified an SF O157:H7 strain that complements the current model of the stepwise evolution from O55:H7 to EHEC O157 in which the hypothetical intermediate between O55:H7 and SF and NSF O157:H7/H^–^ has been unknown (*10**,**12*). As with the highly human pathogenic O157:H7 lineage of EHEC, which is known to reside in cattle, deer, and other ruminants, this intermediate strain was isolated from a deer (*13*). These findings support previous observations (*31**,**32*) and suggest an evolution toward an animal reservoir for O157:H7 soon after O157:H^–^ and O157:H7 divergence. Strain LSU-61 is motile (H-phase 7) and enterohemolysin active (*10*), traits that are typical for NSF O157, further suggesting the intermediate character of LSU-61 between SF and NSF O157. In contrast to the MLST scheme applied from Feng et al. and Lacher et al. (*10**,**33*), our MLST analysis, using the scheme of Wirth et al. (*26*) that analyzes different genes, further corroborates the intermediate character of LSU-61 because it shares the same ST with SF (subgroup B prototype strain) and NSF O157 (subgroup C prototype strains).

Strain LSU-61 does not carry a *stx* gene, but this fact does not contradict our findings because these genes are encoded on bacteriophages that can be acquired and lost (*30**,**34**,**35*), and we do not have evidence of a progenitor to LSU-61 that contains *stx* genes. Although known potential *stx* phage integration sites in O157 were intact, the possibility of a previous *stx* bacteriophage carriage cannot be excluded. If the SF O157:H7 cluster emerged ≈3,000–4,000 years ago (*12*), certain genetic and phenotypic changes (*10*) occurred well before the first descendants of this cluster were isolated and characterized.

Two previous studies (*31**,**32*) reported isolated comparable strains to LSU-61 from (European) red deer, belonging to the same family (*Cervidae*) as white-tailed deer (North America), with comparable phenotypic and genotypic traits. Some of these were SF O157:H7 strains (*stx* negative or positive, β-glucuronidase positive activity) (*31**,**32*). The proof of the existence of SF O157:H7 in a ruminant (deer) host may indicate transfer into animals soon after the 2 (human pathogenic) O157 subgroups B and C emerged. On the basis of shared characteristics with both O157 branches, we suggest strain LSU-61 as a representative of the intermediate cluster complementing the stepwise evolutionary model of EHEC O157. The phylogeny based on either sSNPs or nsSNPs also resulted in a comparable phylogenetic tree with LSU-61 as a member of the progenitor node, underlining its intermediate role.

On the level of gene categories, a higher percentage of sSNPs, though fewer SNPs overall, were observed in the metabolism/housekeeping category compared with the putative metabolism/housekeeping category. The higher rate of nsSNPs in the latter category, resulting in a higher phenotypic diversity, might be explained by uncertain gene categorization because of currently limited knowledge of gene function. Therefore, SNP typing results may help to find genes involved in host–pathogen interactions rather than in metabolism or housekeeping only. SNP data for hypothetical proteins are difficult to interpret because information about their function is too imprecise to enable estimation of the effect of evolutionary pressure.

The fact that 35 of the 38 O157:H7 strains were subgrouped into either cluster 1 or 3 (17 and 18 strains, respectively) shows a certain persistence of these O157:H7 clusters (*29*), characterized by a successful pathogenicity, for example, outbreaks over a broad time frame (*4*). The preponderance of cluster 1 strains has been noted before, as have the paucity of cluster 2 and the diminished proportion of cluster 3 strains in North America (*29*). We observed a higher number of SNPs within the different NSF O157:H7 clusters compared with the few SNPs within restricted SF O157:H^–^ genotypes and a maximum pairwise distance of 2 SNPs (Figure). A reason for this phenomenon may be the different animal host origins for the NSF O157:H7 clade, whereas SF O157:H^–^ are considered to have only 1 main host, humans (*5**,**19*). This high conservation was similarly recognized when multilocus variable-number tandem-repeat analysis was applied (*19*). In this context, certain SNP genotypes may serve to illuminate several strain-specific characteristics, such as increased virulence and other phenotypic traits, as other studies have similarly observed for both SF and NSF O157 (*36**,**37*).

Our results could be interpreted as if C_2_ strain 86–24 is an offshoot of cluster 3, which is in contrast to the established stepwise model of O157. However, we believe that this is an artifact caused by sampling bias of the investigated 92 loci because only 11 backbone SNPs have been found to differentiate cluster 2 and 3 within the whole chromosomal backbone (*12*). One strain (SNPO157_36) did not cluster into any known O157:H7 cluster (Figure).

In summary, our identification of an intermediate member of the EHEC 1 clade complements the current evolutionary model of EHEC O157 by using chromosomal backbone SNP data of a spatiotemporally diverse strain collection. The different levels of genotypic conservation within the subgroups and the animal origin of the intermediate underline the great effect of host–pathogen interaction on the evolution of bacterial species. Future studies should focus on this interaction within both human and animal hosts to understand the evolution and persistence in nature of such human pathogens. The survival of the ancestral pathogen until today suggests that its genetic attributes could be informative in identifying fitness and potentially pathogenic loci.

## Supplementary Material

Technical Appendix This appendix contains a detailed list of the 92 open reading frames (ORFs) (Technical Appendix Table 1) and single nucleotide polymorphisms (SNPs) (Technical Appendix Table 2) of all 50 strains of Enterohemorrhagic Escherichia coli O157 investigated in this study.
