# Supplementary material for: Identification of Intermediate in Evolutionary Model of Enterohemorrhagic Escherichia coli O157
Source: Emerg Infect Dis. 2012 Apr;18(4):582–8. doi: 10.3201/eid1804.111414 (PMC3309690; doi:10.3201/eid1804.111414)
Supplement: Technical Appendix — This appendix contains a detailed list of the 92 open reading frames (ORFs) (Technical Appendix Table 1) and single nucleotide polymorphisms (SNPs) (Technical Appendix Table 2) of all 50 strains of Enterohemorrhagic Escherichia coli O157 investigated in this study. [file 11-1414-Techapp_21p.pdf]

Article DOI: http://dx.doi.org/10.3201/eid1804.111414

# Identification of Intermediate in Evolutionary Model of Enterohemorrhagic *Escherichia coli* O157

## Technical Appendix

This appendix contains a detailed list of the 92 open reading frames (ORFs) (Technical Appendix Table 1) and single nucleotide polymorphisms (SNPs) (Technical Appendix Table 2) of all 50 strains of Enterohemorrhagic *Escherichia coli* O157 investigated in this study. The gene product, functional category, primer sequences, PCR product sizes, and number of base pairs analyzed are given for each ORF, and for each strain, SNP genotype is given at the 111 chromosomal SNP localizations determined in the study. Technical Appendix Table 3 lists all 111 SNPs discovered in this study, with detailed information about the SNP position within the ORF and its absolute position within the reference genome of enterohemorrhagic *E. coli* O157:H7 strain Sakai (GenBank accession no. NC_002695). The reference nucleotide, its variant, and the corresponding codons are listed in addition to the codon effect (synonymous or non-synonymous mutation).

Technical Appendix Table 1. Detailed list of the 92 ORFs investigated

| ORF designation | Gene Product | Gene Category |
|---|---|---|
| ECs0002 | bifunctional aspartokinase I/homeserine dehydrogenase I | metabolism/housekeeping |
| ECs0076 | isopropylmalate isomerase large subunit | metabolism/housekeeping |
| ECs0093 | cell division protein FtsW | putative function based on homology |
| ECs0100 | UDP-3-O-[3-hydroxymyristoyl] N-acetylglucosamine deacetylase | putative function based on homology |
| ECs0204 | putative transcriptional regulator | putative function based on homology |
| ECs0406 | acetaldehyde dehydrogenase | metabolism/housekeeping |
| ECs0444 | fructokinase | metabolism/housekeeping |
| ECs0502 | putative multidrug transporter membrane\ATP-binding components | putative function based on homology |
| ECs0523 | DNA polymerase III subunits gamma and tau | metabolism/housekeeping |
| ECs0530 | inosine-guanosine kinase | putative function based on homology |
| ECs0552 | putative protease | putative function based on homology |
| ECs0614 | phenylalanine transporter | putative function based on homology |
| ECs0615 | putative transport | putative function based on homology |
| ECs0647 | putative oxidoreductase | putative function based on homology |
| ECs0745 | type II citrate synthase | metabolism/housekeeping |
| ECs0905 | molybdopterin biosynthesis protein MoeA | putative function based on homology |
| ECs0917 | putative dehydrogenase | putative function based on homology |
| ECs0955 | putative dTDP-glucose enzyme | putative function based on homology |
| ECs0983 | putative MFS family transporter protein | putative function based on homology |
| ECs0998 | tetraacyldisaccharide 4'-kinase | metabolism/housekeeping |
| ECs1044 | hypothetical protein | Hypothetical protein |
| ECs1046 | DNA helicase IV | putative function based on homology |
| ECs1137 | cryptic autophosphorylating protein tyrosine kinase Etk | putative function based on homology |
| ECs1148 | hybrid sensory histidine kinase TorS | putative function based on homology |
| ECs1721 | calcium/sodium:proton antiporter | putative function based on homology |
| ECs1729 | nitrate reductase 1 alpha subunit | putative function based on homology |
| ECs1741 | bifunctional acetaldehyde-CoA/alcohol dehydrogenase | putative function based on homology |
| ECs1833 | tryptophan synthase subunit beta | metabolism/housekeeping |
| ECs1871 | peptide transport-like protein | putative function based on homology |
| ECs1878 | putative oxidoreductase | putative function based on homology |
| ECs2022 | glyceraldehyde 3-phosphate dehydrogenase C | putative function based on homology |
| ECs2045 | putative ATP-binding component of a transport system | putative function based on homology |
| ECs2087 | putative ATP-binding component of a transport system | putative function based on homology |
| ECs2131 | glutaminase | metabolism/housekeeping |
| ECs2320 | hypothetical protein | Hypothetical protein |
| ECs2406 | hypothetical protein | Hypothetical protein |
| ECs2429 | 6-phosphofructokinase 2 | metabolism/housekeeping |
| ECs2435 | part of a kinase | putative function based on homology |
| ECs2454 | bifunctional succinylornithine transaminase/acetylornithine transaminase | putative function based on homology |
| ECs2488 | glyceraldehyde-3-phosphate dehydrogenase | putative function based on homology |
| ECs2582 | biotin sulfoxide reductase 2 | putative function based on homology |
| ECs2583 | putative cytochrome C-type protein | putative function based on homology |
| ECs2589 | flagellar biosynthesis protein FlhA | putative function based on homology |
| ECs3043 | DNA-binding transcriptional regulator GalS | putative function based on homology |
| ECs3059 | fructose-specific PTS system IIBC component | putative function based on homology |
| ECs3076 | putative ATP-dependent helicase | putative function based on homology |
| ECs3076 | putative ATP-dependent helicase | putative function based on homology |
| ECs3207 | 3-oxoacyl-(acyl carrier protein) synthase I | metabolism/housekeeping |
| ECs3225 | 3-ketoacyl-CoA thiolase | metabolism/housekeeping |
| ECs3228 | hypothetical protein | Hypothetical protein |
| ECs3231 | putative prophage Sf6-like integrase | putative function based on homology |
| ECs3246 | multidrug resistance protein Y | putative function based on homology |
| ECs3300 | N-acetylmuramic acid phosphotransfer permease | putative function based on homology |
| ECs3377 | 4-hydroxy-3-methylbut-2-en-1-yl diphosphate synthase | putative function based on homology |
| ECs3378 | cytoskeletal protein RodZ | putative function based on homology |
| ECs3464 | phospho-2-dehydro-3-deoxyheptonate aldolase | metabolism/housekeeping |
| ECs3564 | D-arabinose 5-phosphate isomerase | putative function based on homology |
| ECs3639 | phosphopyruvate hydratase | metabolism/housekeeping |
| ECs3676 | exonuclease V subunit alpha | putative function based on homology |
| ECs3746 | phenylhydantoinase | metabolism/housekeeping |
| ECs3751 | putative selenate reductase subunit YgfK | putative function based on homology |
| ECs3773 | 6-phospho-beta-glucosidase BglA | putative function based on homology |
| ECs3810 | transketolase | metabolism/housekeeping |
| ECs3833 | L-asparaginase II | metabolism/housekeeping |
| ECs3839 | murein transglycosylase C | putative function based on homology |
| ECs3990 | hypothetical protein | Hypothetical protein |
| ECs3994 | putative formate acetyltransferase 3 | putative function based on homology |

| ORF designation | Gene Product | Gene Category |
|---|---|---|
| ECs4047 | tRNA pseudouridine synthase B | putative function based on homology |
| ECs4109 | malate dehydrogenase | metabolism/housekeeping |
| ECs4130 | sodium/panthothenate symporter | putative function based on homology |
| ECs4204 | putative hydrolase | putative function based on homology |
| ECs4226 | tryptophanyl-tRNA synthetase | metabolism/housekeeping |
| ECs4259 | maltodextrin phosphorylase | putative function based on homology |
| ECs4265 | putative 2-component regulator | putative function based on homology |
| ECs4359 | putative ATP-binding component of a transport system | putative function based on homology |
| ECs4364 | hypothetical protein | Hypothetical protein |
| ECs4411 | endo-1,4-D-glucanase | metabolism/housekeeping |
| ECs4418 | hypothetical protein | Hypothetical protein |
| ECs4424 | peptide ABC transporter | putative function based on homology |
| ECs4454 | periplasmic alpha-amylase precursor | putative function based on homology |
| ECs4493 | putative glycosyl transferase | putative function based on homology |
| ECs4514 | bifunctional phosphopantothenoylcysteine decarboxylase/phosphopantothenate synthase | metabolism/housekeeping |
| ECs4601 | hypothetical protein | Hypothetical protein |
| ECs4689 | potassium transport protein Kup | putative function based on homology |
| ECs4711 | ATP-dependent DNA helicase Rep | putative function based on homology |
| ECs4718 | lipopolysaccharide biosynthesis protein WzzE | putative function based on homology |
| ECs4841 | 6-phosphofructokinase | metabolism/housekeeping |
| ECs4861 | DNA-binding transcriptional regulator CytR | putative function based on homology |
| ECs4872 | hypothetical protein | Hypothetical protein |
| ECs4920 | uroporphyrinogen decarboxylase | metabolism/housekeeping |
| ECs5024 | glycerol-3-phosphate acyltransferase | metabolism/housekeeping |
| ECs5210 | UDP-N-acetylmuramate:L-alanyl-gamma-D-glutamyl- meso-diaminopimelat e ligase | putative function based on homology |
| ECs5222 | aspartate carbamoyltransferase catalytic subunit | metabolism/housekeeping |

| ORF designation | Forward Primer | Sequence of Forward Primer (5'-3') | Reverse Primer | Sequence of Reverse Primer (5'-3') | Amplicon size, bp | No. bp analyzed |
|---|---|---|---|---|---|---|
| ECs0002 | 0002_1558_F | GCATGGCCTAAATCTGGAAA | 0002_2214_R | AGCGCAAAACTTTTCCTTCA | 695 | 593 |
| ECs0076 | 0076_42_F | GAAATGGCCGGTCACAGC | 0076_682_R | CACCTGACGAAACCACCTTT | 677 | 573 |
| ECs0093 | 0093_293_F | GCGTTTATTCTGGCGATCAT | 0093_915_R | CACCGACATACCCCAGTTCT | 661 | 560 |
| ECs0100 | 0100_276_F | GCTTGGGCATCGATAACATT | 0100_851_R | GTCGTCCTGGAAGGTCACAT | 614 | 526 |
| ECs0204 | 0204_40_F | CTCCGGGAGCTGTTTTTACA | 0204_693_R | TCGCGTACAGTCAATTTTGC | 692 | 593 |
| ECs0406 | 0406_61_F | TGGCACCGATCTGATGATTA | 0406_676_R | GCCTCAGCCATTTCATTGAT | 654 | 554 |
| ECs0444 | 0444_203_F | CTGGCTCAATTTCGCCTTAC | 0444_781_R | AACTGCGGAACCGTTTGATA | 607 | 500 |
| ECs0502 | 0502_115_F | ACTGGTTCCACCAAAAGTGG | 0502_715_R | TCGAAACGAGCATCAATACG | 639 | 554 |
| ECs0523 | 0523_843_F | GTATCGAGTGGGAAGCGTTG | 0523_1447_R | TCTTTTTGCTGCATCACTGG | 643 | 542 |
| ECs0530 | 0530_238 | CGAAGCGCTTTATCAGGAAC | 0530_899_R | ATGCTGGGTTTTACGTTTCG | 700 | 607 |
| ECs0552 | 0552_265_F | GCGCTTCAGCCTGTAAAAAC | 0552_885_R | CCCGATTCTCATTTTTGTCG | 659 | 566 |
| ECs0614 | 0614_42_F | CGGAAGATACTGCGTCGAAT | 0614_621_R | ACAAAATCAGCCCATTCCAG | 618 | 528 |
| ECs0615 | 0615_140_F | TTCATATTCCAGCCACACCA | 0615_769_R | TTTTGATGATCTCGCTGCTG | 668 | 570 |
| ECs0647 | 0647_31_F | GACTTATCACGGCCCACATC | 0647_654_R | CAAGCGGTAGGGATGATGA | 661 | 558 |
| ECs0745 | 0745_309_F | GTGCCCAGCTCTTTCAGAAC | 0745_942_R | GACCCGTCATACCATGATCC | 672 | 571 |
| ECs0905 | 0905_574_F | CGCGGATAATCCCTAAGTTG | 0905_1187_R | GACACCGCGCTTAATGAGAT | 652 | 545 |
| ECs0917 | 0917_107_F | CTGCAAGACAAACTCGACCA | 0917_763_R | TAGTTGATTCCCCAGGTTGC | 695 | 586 |
| ECs0955 | 0955_366_F | TGCCACAAAATATTGCCAAA | 0955_973_R | TAATCGTTGGTGCAGGTTCA | 646 | 540 |
| ECs0983 | 0983_281_F | CTTGGCCTGATGATTGGTTT | 0983_898_R | GCCACCGGATATAGCGTAAA | 656 | 571 |
| ECs0998 | 0998_81_F | GGTTGTATGGCCTGGTGAGT | 0998_673_R | TGTTCAAGCTGAGCAACGTC | 631 | 527 |
| ECs1044 | 1044_19_F | TTACGCCGAAACAGGAGTT | 1044_594_R | CGATACGCTGATTGGTTGTG | 613 | 523 |
| ECs1046 | 1046_155_F | ATTCACTGTAAGCGCGGTCT | 1046_730_R | CCAAACGCCAGCAATAAAAT | 614 | 516 |
| ECs1137 | 1137_1518_F | CCGCTTTCTTTACTGCGTTC | 1137_2111_R | ATCGATCTGCTTCGTCTGGT | 632 | 540 |
| ECs1148 | 1148_1491_F | TCCTGCAATTCAGCTGTACG | 1148_2089_R | TGCAGCAAATGGTGATGAAT | 637 | 544 |
| ECs1721 | 1721_338_F | GAAGCCAGTAAAGGCGACAG | 1721_993_R | GGGAAGCAGCCAGACACTAC | 694 | 597 |
| ECs1729 | 1729_484_F | CGGTGGTTTTGTTCGTTCTT | 1729_1102_R | TTCCATTCCGGATTGTTTTC | 657 | 569 |
| ECs1741 | 1741_406_F | TGAATGCAGTCTGCTTGGTC | 1741_982_R | ACGAACATCCGGAAACTCAC | 615 | 519 |
| ECs1833 | 1833_326_F | TTCTTCAATCTGCCCGTCTT | 1833_898_R | GTCAGGCTTTACTGGCGAAG | 611 | 516 |
| ECs1871 | 1871_401_F | CTGAAAGCGACCTTCTACCG | 1871_986_R | GGCACTGGACCGTATCAGTT | 624 | 534 |
| ECs1878 | 1878_393_F | TGTTTGTGGCGATGAATGAT | 1878_976_R | ATTTTCACGCCCTTCAACTG | 622 | 517 |
| ECs2022 | 2022_36_F | TTTTCGAGGGTGCGAATAAG | 2022_645_R | CGCGAAGAAGGTGTTGATTT | 648 | 552 |
| ECs2045 | 2045_191_F | GGCGGGGCTATCTCTATCTT | 2045_793_R | AGTTCACCAGGGGTGTTGAG | 641 | 536 |
| ECs2087 | 2087_281_F | CACAATCTGCCCGAGATACA | 2087_893_R | TAACGTTACGCGACGTCCA | 650 | 558 |
| ECs2131 | 2131_76_F | CAAGCGAGTTACCTGCATCA | 2131_736_R | CGCAAGAACGTTTTTCCATT | 699 | 596 |
| ECs2320 | 2320_280_F | TCAGAGCGTCATCTTCAACG | 2320_891_R | CATCAATTTGGCCGACTTTT | 650 | 553 |
| ECs2406 | 2406_587_F | GGCCTCACACCAGAACAGAT | 2406_1202_R | CATGATCATTTTGCGTACCG | 654 | 564 |
| ECs2429 | 2429_315_F | AAGCAAGCGGTGAGCAGTAT | 2429_882_R | TATCGTCATGGGAGCACAGA | 606 | 503 |

| ORF designation | Forward Primer | Sequence of Forward Primer (5'-3') | Reverse Primer | Sequence of Reverse Primer (5'-3') | Amplicon size, bp | No. bp analyzed |
|---|---|---|---|---|---|---|
| ECs2435 | 2435_40_F | GAACATCGTGGTGTTCGTTG | 2435_666_R | GTTTCATCACCCAGCTTTGC | 665 | 572 |
| ECs2454 | 2454_38_F | AACAAAGTGTTCGCAAGCAA | 2454_657_R | CTCTACCTGTGCGGTGATTG | 658 | 547 |
| ECs2488 | 2488_310_F | TGGTCTGTTCCTGACTGACG | 2488_936_R | AGTAACCGGTTTCGTTGTCG | 665 | 561 |
| ECs2582 | 2582_278_F | GTTCCAGACACGGACCAGAT | 2582_887_R | TTTGAGCGCAATGACTTGAC | 648 | 551 |
| ECs2583 | 2583_495_F | TCGCCTATGCGAGAAGTTTT | 2583_1066_R | CGCATTGGGTTATTGTTTTTG | 611 | 517 |
| ECs2589 | 2589_1054_F | CGGCATTATTCTCTGCCATT | 2589_1706_R | GTTGGTGGCAATTTCGCTAT | 691 | 589 |
| ECs3043 | 3043_240_F | TCATCGAAACCGATGATTGA | 3043_822_R | GGACGTTTCTGATGCGTTTT | 621 | 530 |
| ECs3059 | 3059_51_F | GGACGTTTCAGGAAGGCATA | 3059_669_R | GCAACTGAAACTGCCACAGA | 657 | 570 |
| ECs3076 | 3076a_179_F | GTGCTGGCACACGTTAAAGA | 3076a_834_R | GGGTGTCGCCAGTAATCAGT | 694 | 564 |
| ECs3076 | 3076b_1147_F | CAACGTTCCGGTACAGGTTT | 3076b_1722_R | CGACGAAAACGACCTTCG | 612 | 524 |
| ECs3207 | 3207_386_F | CATCGCCATCTGCATACAAC | 3207_1043_R | CTGGATACCACTGGCCTCAT | 696 | 578 |
| ECs3225 | 3225_63_F | GCACAGGCGGTAACTAAACC | 3225_647_R | GCATGGTCAGACGGAAAACT | 623 | 533 |
| ECs3228 | 3228_189_F | CCATACGAGGAAACGACGAT | 3228_808_R | CGAAATCCTTTTTGAATGTGG | 658 | 566 |
| ECs3231 | 3231_570_F | ACCTTGCTGAAGCCATGAAG | 3231_1132_R | ACCTTCCCATCAAGCCAGTC | 601 | 518 |
| ECs3246 | 3246_302_F | CGACGTACCAACTGATCCTG | 3246_923_R | GACTTCACCGGTCAAAATGAA | 661 | 558 |
| ECs3300 | 3300_365_F | AAATTCGCCACCATCTTCAC | 3300_983_R | GCTGTTGAATCCTTGGCTGT | 657 | 567 |
| ECs3377 | 3377_470_F | ACCAATGGCGGATTTTACTG | 3377_1098_R | GCATAACCAGGCTCCAATTC | 667 | 563 |
| ECs3378 | 3378_242_F | CCAGCAATCGGCAGTAAAGT | 3378_836_R | GCCGATCTTGCTTCAACATT | 633 | 534 |
| ECs3464 | 3464_407_F | CGGATTCCCCTGAGTTTGTA | 3464_1003_R | TAATGACTCCGGCACAACTG | 635 | 546 |
| ECs3564 | 3564_287_F | CGCGATGTGATGCTGTTTAT | 3564_898_R | AGTTTGCCGTTTTCATCCAC | 650 | 552 |
| ECs3639 | 3639_69_F | CCCAGAGCTTCTTCGATACG | 3639_681_R | AGGCATGAACACTGCTGTTG | 651 | 562 |
| ECs3676 | 3676_1166_F | ATCGGTCAGCGGTAACTGTC | 3676_1784_R | GAAGCTGTGGAGCACAAACA | 657 | 574 |
| ECs3746 | 3746_719_F | CCGCTCTATATCGTGCACCT | 3746_1302_R | AGGTGCCGTCACAGAAAATC | 622 | 530 |
| ECs3751 | 3751_2250_F | ATGCTGATGGCACCTTAACC | 3751_2873_R | CAGGCTGAAAACGGTGATTT | 662 | 559 |
| ECs3773 | 3773_726_F | TGAAAGTCGGCTGTATGCTG | 3773_1351_R | ATATCGCCAGTACCGTCGTC | 664 | 519 |
| ECs3810 | 3810_1293_F | AGGGACGGTTTGTCAGTCAC | 3810_1932_R | GAGCATGGACGCAGTACAGA | 678 | 533 |
| ECs3833 | 3833_299_F | CGCATCTACCAGGGCTTTAG | 3833_913_R | GTGACTCCGCAACCAAATCT | 653 | 556 |
| ECs3839 | 3839_474_F | CAAGCAACTTCGCGGATTAT | 3839_1047_R | ATTTTTGCGCGGTATTCACT | 612 | 319 |
| ECs3990 | 3990_378_F | CGCGGTAACTGGTTATGGAT | 3990_1018_R | GGCTGGAAGTGCTGAAAGAC | 679 | 571 |
| ECs3994 | 3994_524_F | TGGCAGCGCTTTAATTTTCT | 3994_1136_R | TCGGAAGAATTACCGATTGC | 651 | 545 |
| ECs4047 | 4047_149_F | TGTACGAACCGGGTTACCAT | 4047_743_R | GCGACGAAGTTTTCCCAGTA | 633 | 529 |
| ECs4109 | 4109_39_F | GCCCAGGGCGATATCTTT | 4109_666_R | GGATCGTTCCGACCTGTTTA | 664 | 572 |
| ECs4130 | 4130_367_F | CCGCTACCAGAGTCGTCTTC | 4130_1002_R | GCAATTGGGCGTTAATTGTC | 674 | 584 |
| ECs4204 | 4204_235_F | ACCTCGTCTGGTGGTGTTTC | 4204_806_R | AATAATCAGCGTCGGTTTGG | 610 | 502 |
| ECs4226 | 4226_37_F | AACCAATCGCTTCGTACACC | 4226_671_R | CGCATGACGCAGTTTAAAGA | 673 | 574 |
| ECs4259 | 4259_288_F | TTCAATACTGCGTCCAGCAC | 4259_884_R | CAGCAATATCGCGAGATCAA | 635 | 533 |
| ECs4265 | 4265_233_F | GTGGTGAGCGTTGAGATTGA | 4265_823_R | GCGCTGCGTAATAATCCTTC | 629 | 528 |
| ECs4359 | 4359_780_F | ATCCAGACACCGAGTTCCAC | 4359_1384_R | TGTTCTGGCAGTTGATGGTC | 643 | 540 |
| ECs4364 | 4364_20_F | AGCACGACTTCGCTGCAA | 4364_662_R | AAGATTGCCGAACAATTTGG | 679 | 583 |
| ECs4411 | 4411_428_F | TTTTAGCTGCCAGCCTTTGT | 4411_1047_R | GGCTGCCTTTAGTGTTCAGG | 658 | 541 |
| ECs4418 | 4418_150_F | CGCTCCTCAATTTGGTGTTT | 4418_781_R | TCAATATCGAAATGCGACCA | 670 | 565 |
| ECs4424 | 4424_906_F | GTATCGATCTGCGGTTTGGT | 4424_1564_R | GGATGCTGAAGCTTGGTCTC | 697 | 599 |
| ECs4454 | 4454_42_F | TGACACTCCTTCCTGGCTTC | 4454_639_R | TTTCCGCCATACCGTCTTTA | 636 | 546 |
| ECs4493 | 4493_361_F | GCAACCGACTCACTGACGTA | 4493_956_R | TATAATGCGGGCGATGATTT | 634 | 544 |
| ECs4514 | 4514_239_F | GGCCATATTGAGCTGGGTAA | 4514_864_R | TGGCCTGCTTTTTGATTTTC | 664 | 547 |
| ECs4601 | 4601_38_F | CGACTGCCACACCAGAAGTA | 4601_602_R | TTTTTGCTCACGCTATTTCG | 603 | 518 |
| ECs4689 | 4689_322_F | GCTGGTGATTATGGGGCTAA | 4689_979_R | TCCATTTCGGAGGTGTGAAT | 696 | 547 |
| ECs4711 | 4711_22_F | GTCTAAACCCCGGCCAAC | 4711_654_R | CATACTGGCTGGTGTTGGTG | 669 | 572 |
| ECs4718 | 4718_366_F | GAGAGTTCTGGCTGCAAACC | 4718_965_R | AATCATCAGGAAGGCACGAC | 638 | 518 |
| ECs4841 | 4841_181_F | CCGTTACAGCGTTTCTGACA | 4841_786_R | CCATACGGGAAGCCAGAATA | 644 | 555 |
| ECs4861 | 4861_177_F | TCACAAAATTGCGTCAGGTC | 4861_829_R | GCAACGTCAAGCGTAATGAA | 691 | 595 |
| ECs4872 | 4872_234_F | CCTTAGCCATTGCCCTGTTA | 4872_828_R | GCACAATCAGCACAATACCG | 633 | 543 |
| ECs4920 | 4920_376_F | TGAACTGGGGTACGTGATGA | 4920_965_R | GCCGTGACCAAGGTTAAAGA | 628 | 541 |
| ECs5024 | 5024_1136_F | GGCGTAAGTACCCACTTCCA | 5024_1730_R | ATTGCCAAAGCGGTAGAAGA | 633 | 515 |
| ECs5210 | 5210_149_F | AAGCAAGGCATTGAGCTGAT | 5210_772_R | TCCAGCAAAACTTCCCATTC | 662 | 566 |
| ECs5222 | 5222_284_F | GTCAACTTCCGCCATCACTT | 5222_913_R | TGGCTAATCCGCTATATCAGAAA | 671 | 575 |

Technical Appendix Table 2. SNP list of all 50 strains investigated

| | Locus tag (ORF designation) SNP position within ORF | | | | | |
|---|---|---|---|---|---|---|
| Strain ID | ECs0076.SNP-1212 | ECs0076.SNP-1164 | ECs0093.SNP-687 | ECs0100.SNP-406 | ECs0204.SNP-573 | ECs0406.SNP-494 |
| 493/89 | C | C | C | G | C | C |
| 86-24 | C | T | C | G | C | C |
| 87-14 | C | T | C | G | C | C |
| CB9615 | T | C | C | A | C | T |
| EC4115 | C | T | C | G | C | C |
| EDL933 | C | T | C | G | C | C |
| LSU-61 | C | C | C | G | C | C |
| Sakai | C | T | C | G | C | C |
| TB182A | T | C | C | A | C | C |
| TW14359 | C | T | C | G | C | C |
| TW14588 | C | T | C | G | C | C |
| SNPO157_01 | C | T | C | G | C | C |
| SNPO157_02 | C | T | T | G | T | C |
| SNPO157_03 | C | C | C | G | C | C |
| SNPO157_04 | C | T | C | G | C | C |
| SNPO157_05 | C | T | C | G | C | C |
| SNPO157_06 | C | T | C | G | C | C |
| SNPO157_07 | C | T | T | G | C | C |
| SNPO157_08 | C | T | C | G | C | C |
| SNPO157_09 | C | C | C | G | C | C |
| SNPO157_10 | C | T | C | G | C | C |
| SNPO157_11 | C | C | C | G | C | C |
| SNPO157_12 | C | C | C | G | C | C |
| SNPO157_13 | C | T | T | G | C | C |
| SNPO157_14 | C | T | C | G | C | C |
| SNPO157_15 | C | T | C | G | C | C |
| SNPO157_16 | C | T | C | G | C | C |
| SNPO157_17 | C | T | C | G | C | C |
| SNPO157_18 | C | T | C | G | C | C |
| SNPO157_19 | C | T | C | G | C | C |
| SNPO157_20 | C | T | C | G | C | C |
| SNPO157_21 | C | C | C | G | C | C |
| SNPO157_22 | C | T | C | G | C | C |
| SNPO157_23 | C | T | C | G | C | C |
| SNPO157_24 | C | T | C | G | C | C |
| SNPO157_25 | C | T | C | G | C | C |
| SNPO157_26 | C | T | C | G | C | C |
| SNPO157_27 | C | T | C | G | C | C |
| SNPO157_28 | C | T | C | G | C | C |
| SNPO157_29 | C | T | C | G | C | C |
| SNPO157_30 | C | T | C | G | C | C |
| SNPO157_31 | C | T | C | G | C | C |
| SNPO157_32 | C | T | C | G | C | C |
| SNPO157_33 | C | T | C | G | C | C |
| SNPO157_34 | C | C | C | G | C | C |
| SNPO157_35 | C | C | C | G | C | C |
| SNPO157_36 | C | C | C | G | C | C |
| SNPO157_37 | C | C | C | G | C | C |
| SNPO157_38 | C | T | C | G | C | C |
| SNPO157_39 | C | C | C | G | C | C |

| | Locus tag (ORF designation) SNP position within ORF | | | | | | |
|---|---|---|---|---|---|---|---|
| Strain ID | ECs0444.SNP-260 | ECs0502.SNP-342 | ECs0523.1134 | ECs0552.SNP-186 | ECs0614.SNP-138 | ECs0614.SNP-283 | ECs0647.SNP-216 |
| 493/89 | C | T | C | C | C | A | C |
| 86-24 | C | T | C | C | C | T | T |
| 87-14 | C | T | C | C | T | T | T |
| CB9615 | C | G | C | C | C | T | C |
| EC4115 | C | T | C | C | C | T | T |
| EDL933 | C | T | C | C | C | T | T |
| LSU-61 | C | T | T | C | C | T | C |
| Sakai | C | T | C | C | C | T | T |
| TB182A | C | G | C | C | C | T | C |
| TW14359 | C | T | C | C | C | T | T |
| TW14588 | C | T | C | C | C | T | T |

| | Locus tag (ORF designation) SNP position within ORF | | | | | | |
|---|---|---|---|---|---|---|---|
| Strain ID | ECs0444.SNP-260 | ECs0502.SNP-342 | ECs0523.1134 | ECs0552.SNP-186 | ECs0614.SNP-138 | ECs0614.SNP-283 | ECs0647.SNP-216 |
| SNPO157_01 | C | T | C | C | T | T | T |
| SNPO157_02 | C | T | C | C | C | T | T |
| SNPO157_03 | C | T | C | C | C | A | C |
| SNPO157_04 | C | T | C | C | T | T | T |
| SNPO157_05 | C | T | C | T | C | T | T |
| SNPO157_06 | C | T | C | C | C | T | T |
| SNPO157_07 | C | T | C | C | C | T | T |
| SNPO157_08 | C | T | C | C | T | T | T |
| SNPO157_09 | C | T | C | C | C | A | C |
| SNPO157_10 | C | T | C | C | T | T | T |
| SNPO157_11 | C | T | C | C | C | A | C |
| SNPO157_12 | C | T | C | C | C | T | C |
| SNPO157_13 | C | T | C | C | C | T | T |
| SNPO157_14 | C | T | C | C | T | T | T |
| SNPO157_15 | C | T | C | C | T | T | T |
| SNPO157_16 | C | T | C | C | T | T | T |
| SNPO157_17 | C | T | C | C | T | T | T |
| SNPO157_18 | C | T | C | C | C | T | T |
| SNPO157_19 | C | T | C | C | T | T | T |
| SNPO157_20 | C | T | C | C | C | T | T |
| SNPO157_21 | C | T | C | C | C | A | C |
| SNPO157_22 | C | T | C | C | C | T | T |
| SNPO157_23 | C | T | C | C | C | T | T |
| SNPO157_24 | T | T | C | C | T | T | T |
| SNPO157_25 | C | T | C | C | C | T | T |
| SNPO157_26 | C | T | C | C | T | T | T |
| SNPO157_27 | C | T | C | C | C | T | T |
| SNPO157_28 | C | T | C | C | C | T | T |
| SNPO157_29 | C | T | C | C | T | T | T |
| SNPO157_30 | C | T | C | C | C | T | T |
| SNPO157_31 | C | T | C | C | C | T | T |
| SNPO157_32 | C | T | C | C | C | T | T |
| SNPO157_33 | C | T | C | C | C | T | T |
| SNPO157_34 | C | T | C | C | C | A | C |
| SNPO157_35 | C | T | C | C | C | A | C |
| SNPO157_36 | C | T | C | C | C | T | T |
| SNPO157_37 | C | T | C | C | C | A | C |
| SNPO157_38 | C | T | C | C | C | T | T |
| SNPO157_39 | C | T | C | C | C | A | C |

| | Locus tag (ORF designation) SNP position within ORF | | | | | |
|---|---|---|---|---|---|---|
| Strain ID | ECs0647.SNP-306 | ECs0647.SNP-556 | ECs0905.SNP-453 | ECs0905.SNP-217 | ECs0917.SNP-578 | ECs0955.SNP-745 |
| 493/89 | C | C | C | C | C | C |
| 86-24 | C | C | C | G | C | C |
| 87-14 | C | C | C | G | C | C |
| CB9615 | C | C | T | G | C | C |
| EC4115 | T | C | C | G | C | C |
| EDL933 | C | C | C | G | C | C |
| LSU-61 | C | C | C | G | C | A |
| Sakai | C | C | C | G | C | C |
| TB182A | C | C | T | G | C | C |
| TW14359 | T | C | C | G | C | C |
| TW14588 | C | C | C | G | C | C |
| SNPO157_01 | C | C | C | G | C | C |
| SNPO157_02 | T | C | C | G | C | C |
| SNPO157_03 | C | C | C | C | C | C |
| SNPO157_04 | C | C | C | G | C | C |
| SNPO157_05 | C | C | C | G | C | C |
| SNPO157_06 | C | C | C | G | C | C |
| SNPO157_07 | T | C | C | G | C | C |
| SNPO157_08 | C | C | C | G | C | C |
| SNPO157_09 | C | C | C | C | C | C |
| SNPO157_10 | C | C | C | G | C | C |
| SNPO157_11 | C | C | C | C | C | C |
| SNPO157_12 | C | C | C | C | C | C |

| Strain ID | Locus tag (ORF designation) SNP position within ORF | | | | | |
|---|---|---|---|---|---|---|
| | ECs0647.SNP-306 | ECs0647.SNP-556 | ECs0905.SNP-453 | ECs0905.SNP-217 | ECs0917.SNP-578 | ECs0955.SNP-745 |
| SNPO157_13 | T | C | C | G | C | C |
| SNPO157_14 | C | C | C | G | C | C |
| SNPO157_15 | C | C | C | G | C | C |
| SNPO157_16 | C | C | C | G | C | C |
| SNPO157_17 | C | C | C | G | C | C |
| SNPO157_18 | C | C | C | G | C | C |
| SNPO157_19 | C | C | C | G | C | C |
| SNPO157_20 | C | C | C | G | C | C |
| SNPO157_21 | C | C | C | C | C | C |
| SNPO157_22 | C | C | C | G | C | C |
| SNPO157_23 | C | C | C | G | C | C |
| SNPO157_24 | C | C | C | G | C | C |
| SNPO157_25 | C | C | C | G | C | C |
| SNPO157_26 | C | C | C | G | C | C |
| SNPO157_27 | C | C | C | G | C | C |
| SNPO157_28 | C | C | C | G | T | C |
| SNPO157_29 | C | C | C | G | C | C |
| SNPO157_30 | C | C | C | G | C | C |
| SNPO157_31 | C | C | C | G | T | C |
| SNPO157_32 | C | C | C | G | C | C |
| SNPO157_33 | C | C | C | G | T | C |
| SNPO157_34 | C | C | C | C | C | C |
| SNPO157_35 | C | C | C | C | C | C |
| SNPO157_36 | C | G | C | G | C | C |
| SNPO157_37 | C | C | C | C | C | C |
| SNPO157_38 | C | C | C | G | C | C |
| SNPO157_39 | C | C | C | C | C | C |

| Strain ID | Locus tag (ORF designation) SNP position within ORF | | | | | |
|---|---|---|---|---|---|---|
| | ECs0955.SNP-719 | ECs0998.SNP-375 | ECs1044.SNP-2080 | ECs1044.SNP-1773 | ECs1046.SNP-319 | ECs1046.SNP-358 |
| 493/89 | C | C | A | C | C | C |
| 86-24 | C | T | A | C | C | C |
| 87-14 | C | C | A | C | C | C |
| CB9615 | C | C | A | C | C | A |
| EC4115 | C | C | G | C | C | C |
| EDL933 | C | C | A | T | C | C |
| LSU-61 | C | C | A | C | C | C |
| Sakai | C | C | A | C | C | C |
| TB182A | T | C | A | C | C | A |
| TW14359 | C | C | G | C | C | C |
| TW14588 | C | C | A | C | C | C |
| SNPO157_01 | C | C | A | C | C | C |
| SNPO157_02 | C | C | A | C | C | C |
| SNPO157_03 | C | C | A | C | C | C |
| SNPO157_04 | C | C | A | C | C | C |
| SNPO157_05 | C | C | A | C | C | C |
| SNPO157_06 | C | C | A | C | C | C |
| SNPO157_07 | C | C | A | C | C | C |
| SNPO157_08 | C | C | A | C | C | C |
| SNPO157_09 | C | C | A | C | C | C |
| SNPO157_10 | C | C | A | C | C | C |
| SNPO157_11 | C | C | A | C | C | C |
| SNPO157_12 | C | C | A | C | C | C |
| SNPO157_13 | C | C | A | C | C | C |
| SNPO157_14 | C | C | A | C | C | C |
| SNPO157_15 | C | C | A | C | C | C |
| SNPO157_16 | C | C | A | C | C | C |
| SNPO157_17 | C | C | A | C | C | C |
| SNPO157_18 | C | C | A | C | C | C |
| SNPO157_19 | C | C | A | C | C | C |
| SNPO157_20 | C | C | A | C | C | C |
| SNPO157_21 | C | C | A | C | C | C |
| SNPO157_22 | C | C | A | C | C | C |
| SNPO157_23 | C | C | A | C | C | C |
| SNPO157_24 | C | C | A | C | C | C |

| Strain ID | Locus tag (ORF designation) SNP position within ORF | | | | | |
|---|---|---|---|---|---|---|
| | ECs0955.SNP-719 | ECs0998.SNP-375 | ECs1044.SNP-2080 | ECs1044.SNP-1773 | ECs1046.SNP-319 | ECs1046.SNP-358 |
| SNPO157_25 | C | C | A | C | C | C |
| SNPO157_26 | C | C | A | C | C | C |
| SNPO157_27 | C | C | A | C | C | C |
| SNPO157_28 | C | C | A | C | T | C |
| SNPO157_29 | C | C | A | C | C | C |
| SNPO157_30 | C | C | A | C | C | C |
| SNPO157_31 | C | C | A | C | T | C |
| SNPO157_32 | C | C | A | C | C | C |
| SNPO157_33 | C | C | A | C | T | C |
| SNPO157_34 | C | C | A | C | C | C |
| SNPO157_35 | C | C | A | C | C | C |
| SNPO157_36 | C | C | A | C | C | C |
| SNPO157_37 | C | C | A | C | C | C |
| SNPO157_38 | C | C | A | C | C | C |
| SNPO157_39 | C | C | A | C | C | C |

| Strain ID | Locus tag (ORF designation) SNP position within ORF | | | | | |
|---|---|---|---|---|---|---|
| | ECs1137.SNP-524 | ECs1137.SNP-230 | ECs1148.SNP-1014 | ECs1148.SNP-940 | ECs1729.SNP-786 | ECs1741.SNP-2155 |
| 493/89 | A | C | C | A | C | A |
| 86-24 | A | C | C | G | C | G |
| 87-14 | A | C | C | G | C | G |
| CB9615 | G | C | C | G | C | G |
| EC4115 | A | C | C | G | T | G |
| EDL933 | A | C | C | G | C | G |
| LSU-61 | A | T | C | G | C | G |
| Sakai | A | C | C | G | C | G |
| TB182A | G | C | A | G | C | G |
| TW14359 | A | C | C | G | T | G |
| TW14588 | A | C | C | G | C | G |
| SNPO157_01 | A | C | C | G | C | G |
| SNPO157_02 | A | C | C | G | T | G |
| SNPO157_03 | A | C | C | A | C | A |
| SNPO157_04 | A | C | C | G | C | G |
| SNPO157_05 | A | C | C | G | C | G |
| SNPO157_06 | A | C | C | G | C | G |
| SNPO157_07 | A | C | C | G | T | G |
| SNPO157_08 | A | C | C | G | C | G |
| SNPO157_09 | A | C | C | A | C | A |
| SNPO157_10 | A | C | C | G | C | G |
| SNPO157_11 | A | C | C | A | C | A |
| SNPO157_12 | A | C | C | A | C | A |
| SNPO157_13 | A | C | C | G | T | G |
| SNPO157_14 | A | C | C | G | C | G |
| SNPO157_15 | A | C | C | G | C | G |
| SNPO157_16 | A | C | C | G | C | G |
| SNPO157_17 | A | C | C | G | C | G |
| SNPO157_18 | A | C | C | G | C | G |
| SNPO157_19 | A | C | C | G | C | G |
| SNPO157_20 | A | C | C | G | C | G |
| SNPO157_21 | A | C | C | A | C | A |
| SNPO157_22 | A | C | C | G | C | G |
| SNPO157_23 | A | C | C | G | C | G |
| SNPO157_24 | A | C | C | G | C | G |
| SNPO157_25 | A | C | C | G | C | G |
| SNPO157_26 | A | C | C | G | C | G |
| SNPO157_27 | A | C | C | G | C | G |
| SNPO157_28 | A | C | C | G | C | G |
| SNPO157_29 | A | C | C | G | C | G |
| SNPO157_30 | A | C | C | G | C | G |
| SNPO157_31 | A | C | C | G | C | G |
| SNPO157_32 | A | C | C | G | C | G |
| SNPO157_33 | A | C | C | G | C | G |
| SNPO157_34 | A | C | C | A | C | A |
| SNPO157_35 | A | C | C | A | C | A |

| Strain ID | Locus tag (ORF designation) SNP position within ORF | | | | | |
|---|---|---|---|---|---|---|
| | ECs1137.SNP-524 | ECs1137.SNP-230 | ECs1148.SNP-1014 | ECs1148.SNP-940 | ECs1729.SNP-786 | ECs1741.SNP-2155 |
| SNPO157_36 | A | C | C | G | C | G |
| SNPO157_37 | A | C | C | A | C | A |
| SNPO157_38 | A | C | C | G | C | G |
| SNPO157_39 | A | C | C | A | C | A |

| Strain ID | Locus tag (ORF designation) SNP position within ORF | | | | | |
|---|---|---|---|---|---|---|
| | ECs1833.SNP-474 | ECs1871.SNP-1109 | ECs1871.SNP-978 | ECs1878.SNP-829 | ECs2022.SNP-793 | ECs2045.SNP-677 |
| 493/89 | C | G | A | T | T | C |
| 86-24 | C | A | G | G | T | C |
| 87-14 | C | A | G | T | T | C |
| CB9615 | C | G | G | T | T | T |
| EC4115 | C | A | G | T | T | C |
| EDL933 | C | A | G | G | T | C |
| LSU-61 | C | G | G | T | T | C |
| Sakai | C | A | G | G | T | C |
| TB182A | C | G | G | T | T | T |
| TW14359 | C | A | G | T | T | C |
| TW14588 | C | A | G | G | T | C |
| SNPO157_01 | C | A | G | T | T | C |
| SNPO157_02 | C | A | G | T | T | C |
| SNPO157_03 | C | G | A | T | T | C |
| SNPO157_04 | C | A | G | T | T | C |
| SNPO157_05 | C | A | G | G | T | C |
| SNPO157_06 | C | A | G | G | T | C |
| SNPO157_07 | C | A | G | T | T | C |
| SNPO157_08 | C | A | G | T | T | C |
| SNPO157_09 | C | G | A | T | T | C |
| SNPO157_10 | C | A | G | T | T | C |
| SNPO157_11 | C | G | A | T | T | C |
| SNPO157_12 | C | G | A | T | T | C |
| SNPO157_13 | C | A | G | T | T | C |
| SNPO157_14 | C | A | G | T | T | C |
| SNPO157_15 | C | A | G | T | T | C |
| SNPO157_16 | C | A | G | T | T | C |
| SNPO157_17 | C | A | G | T | T | C |
| SNPO157_18 | C | A | G | G | T | C |
| SNPO157_19 | C | A | G | T | T | C |
| SNPO157_20 | C | A | G | G | T | C |
| SNPO157_21 | T | G | A | T | T | C |
| SNPO157_22 | C | A | G | G | T | C |
| SNPO157_23 | C | A | G | G | T | C |
| SNPO157_24 | C | A | G | T | T | C |
| SNPO157_25 | C | A | G | G | T | C |
| SNPO157_26 | C | A | G | T | T | C |
| SNPO157_27 | C | A | G | G | T | C |
| SNPO157_28 | C | A | G | G | T | C |
| SNPO157_29 | C | A | G | T | T | C |
| SNPO157_30 | C | A | G | G | T | C |
| SNPO157_31 | C | A | G | G | T | C |
| SNPO157_32 | C | A | G | G | T | C |
| SNPO157_33 | C | A | G | G | T | C |
| SNPO157_34 | C | G | A | T | T | C |
| SNPO157_35 | C | G | A | T | T | C |
| SNPO157_36 | C | A | G | T | A | C |
| SNPO157_37 | C | G | A | T | T | C |
| SNPO157_38 | C | A | G | G | T | C |
| SNPO157_39 | C | G | A | T | T | C |

| Strain ID | Locus tag (ORF designation) SNP position within ORF | | | | | |
|---|---|---|---|---|---|---|
| | ECs2087.SNP-253 | ECs2320.SNP-486 | ECs2320.SNP-719 | ECs2435.SNP-123 | ECs2435.SNP-458 | ECs2454.SNP-1064 |
| 493/89 | C | G | A | G | A | G |
| 86-24 | T | A | A | G | T | G |

| Strain ID | Locus tag (ORF designation) SNP position within ORF | | | | | |
|---|---|---|---|---|---|---|
| | ECs2087.SNP-253 | ECs2320.SNP-486 | ECs2320.SNP-719 | ECs2435.SNP-123 | ECs2435.SNP-458 | ECs2454.SNP-1064 |
| 87-14 | T | A | T | G | A | A |
| CB9615 | C | G | A | G | A | G |
| EC4115 | T | A | T | A | A | G |
| EDL933 | T | A | A | G | T | G |
| LSU-61 | C | G | A | G | A | G |
| Sakai | T | A | A | G | T | G |
| TB182A | C | G | A | G | A | G |
| TW14359 | T | A | T | A | A | G |
| TW14588 | T | A | A | G | T | G |
| SNPO157_01 | T | A | T | G | A | A |
| SNPO157_02 | T | A | T | G | A | G |
| SNPO157_03 | C | G | A | G | A | G |
| SNPO157_04 | T | A | T | G | A | A |
| SNPO157_05 | T | A | A | G | A | G |
| SNPO157_06 | T | A | A | G | A | G |
| SNPO157_07 | T | A | T | G | A | G |
| SNPO157_08 | T | A | T | G | A | A |
| SNPO157_09 | C | G | A | G | A | G |
| SNPO157_10 | T | A | T | G | A | A |
| SNPO157_11 | C | G | A | G | A | G |
| SNPO157_12 | C | G | A | G | A | G |
| SNPO157_13 | T | A | T | G | A | G |
| SNPO157_14 | T | A | T | G | A | A |
| SNPO157_15 | T | A | T | G | A | A |
| SNPO157_16 | T | A | T | G | A | A |
| SNPO157_17 | T | A | T | G | A | A |
| SNPO157_18 | T | A | A | G | A | G |
| SNPO157_19 | T | A | T | G | A | A |
| SNPO157_20 | T | A | A | G | T | G |
| SNPO157_21 | C | G | A | G | A | G |
| SNPO157_22 | T | A | A | G | A | G |
| SNPO157_23 | T | A | A | G | T | G |
| SNPO157_24 | T | A | T | G | A | A |
| SNPO157_25 | T | A | A | G | A | G |
| SNPO157_26 | T | A | T | G | A | A |
| SNPO157_27 | T | A | A | G | A | G |
| SNPO157_28 | T | A | A | G | A | G |
| SNPO157_29 | T | A | T | G | A | A |
| SNPO157_30 | T | A | A | G | A | G |
| SNPO157_31 | T | A | A | G | A | G |
| SNPO157_32 | T | A | A | G | A | G |
| SNPO157_33 | T | A | A | G | A | G |
| SNPO157_34 | C | G | A | G | A | G |
| SNPO157_35 | C | G | A | G | A | G |
| SNPO157_36 | T | G | A | G | A | G |
| SNPO157_37 | C | G | A | G | A | G |
| SNPO157_38 | T | A | A | G | A | G |
| SNPO157_39 | C | G | A | G | A | G |

| Strain ID | Locus tag (ORF designation) SNP position within ORF | | | | | |
|---|---|---|---|---|---|---|
| | ECs2454.SNP-693 | ECs2582.SNP-2038 | ECs2583.SNP-73 | ECs2589.SNP-804 | ECs2589.SNP-771 | ECs3043.ESNP-721 |
| 493/89 | A | G | G | A | A | C |
| 86-24 | A | G | T | A | A | C |
| 87-14 | A | A | T | A | A | C |
| CB9615 | G | G | G | A | C | C |
| EC4115 | A | G | T | A | A | C |
| EDL933 | A | G | T | A | A | C |
| LSU-61 | A | G | G | C | A | C |
| Sakai | A | G | T | A | A | C |
| TB182A | G | G | G | A | C | C |
| TW14359 | A | G | T | A | A | C |
| TW14588 | A | G | T | A | A | C |
| SNPO157_01 | A | G | T | A | A | C |
| SNPO157_02 | A | G | T | A | A | C |

| Strain ID | Locus tag (ORF designation) SNP position within ORF | | | | | |
|---|---|---|---|---|---|---|
| | ECs2454.SNP-693 | ECs2582.SNP-2038 | ECs2583.SNP-73 | ECs2589.SNP-804 | ECs2589.SNP-771 | ECs3043.ESNP-721 |
| SNPO157_03 | A | G | G | A | A | C |
| SNPO157_04 | A | G | T | A | A | C |
| SNPO157_05 | A | G | T | A | A | C |
| SNPO157_06 | A | G | T | A | A | C |
| SNPO157_07 | A | G | T | A | A | C |
| SNPO157_08 | A | G | T | A | A | C |
| SNPO157_09 | A | G | G | A | A | C |
| SNPO157_10 | A | G | T | A | A | C |
| SNPO157_11 | A | G | G | A | A | C |
| SNPO157_12 | A | G | G | A | A | C |
| SNPO157_13 | A | G | T | A | A | C |
| SNPO157_14 | A | G | T | A | A | C |
| SNPO157_15 | A | G | T | A | A | C |
| SNPO157_16 | A | G | T | A | A | C |
| SNPO157_17 | A | G | T | A | A | C |
| SNPO157_18 | A | G | T | A | A | C |
| SNPO157_19 | A | G | T | A | A | C |
| SNPO157_20 | A | G | T | A | A | C |
| SNPO157_21 | A | G | G | A | A | C |
| SNPO157_22 | A | G | T | A | A | C |
| SNPO157_23 | A | G | T | A | A | C |
| SNPO157_24 | A | G | T | A | A | T |
| SNPO157_25 | A | G | T | A | A | C |
| SNPO157_26 | A | G | T | A | A | C |
| SNPO157_27 | A | G | T | A | A | C |
| SNPO157_28 | A | G | T | A | A | C |
| SNPO157_29 | A | G | T | A | A | C |
| SNPO157_30 | A | G | T | A | A | C |
| SNPO157_31 | A | G | T | A | A | C |
| SNPO157_32 | A | G | T | A | A | C |
| SNPO157_33 | A | G | T | A | A | C |
| SNPO157_34 | A | G | G | A | A | C |
| SNPO157_35 | A | G | G | A | A | C |
| SNPO157_36 | A | G | T | A | A | C |
| SNPO157_37 | A | G | G | A | A | C |
| SNPO157_38 | A | G | T | A | A | C |
| SNPO157_39 | A | G | G | A | A | C |

| Strain ID | Locus tag (ORF designation) SNP position within ORF | | | | | |
|---|---|---|---|---|---|---|
| | ECs3059.SNP-1440 | ECs3076.SNP-311 | ECs3076.SNP-573 | ECs3076.SNP-1413 | ECs3076.SNP-1689 | ECs3207.SNP-291 |
| 493/89 | C | T | T | G | G | C |
| 86-24 | A | T | T | G | G | C |
| 87-14 | A | C | T | G | A | C |
| CB9615 | C | T | T | G | G | A |
| EC4115 | A | T | T | G | G | C |
| EDL933 | A | T | T | G | G | C |
| LSU-61 | C | T | T | G | G | C |
| Sakai | A | T | T | G | G | C |
| TB182A | C | T | G | A | G | A |
| TW14359 | A | T | T | G | G | C |
| TW14588 | A | T | T | G | G | C |
| SNPO157_01 | A | C | T | G | A | C |
| SNPO157_02 | A | T | T | G | G | C |
| SNPO157_03 | C | T | T | G | G | C |
| SNPO157_04 | A | C | T | G | A | C |
| SNPO157_05 | A | T | T | G | G | C |
| SNPO157_06 | A | T | T | G | G | C |
| SNPO157_07 | A | T | T | G | G | C |
| SNPO157_08 | A | T | T | G | G | C |
| SNPO157_09 | C | T | T | G | G | C |
| SNPO157_10 | A | C | T | G | A | C |
| SNPO157_11 | C | T | T | G | G | C |
| SNPO157_12 | C | T | T | G | G | C |
| SNPO157_13 | A | T | T | G | G | C |

| | Locus tag (ORF designation) SNP position within ORF | | | | | |
|---|---|---|---|---|---|---|
| Strain ID | ECs3059.SNP-1440 | ECs3076.SNP-311 | ECs3076.SNP-573 | ECs3076.SNP-1413 | ECs3076.SNP-1689 | ECs3207.SNP-291 |
| SNPO157_14 | A | C | T | G | A | C |
| SNPO157_15 | A | C | T | G | A | C |
| SNPO157_16 | A | C | T | G | A | C |
| SNPO157_17 | A | C | T | G | A | C |
| SNPO157_18 | A | T | T | G | G | C |
| SNPO157_19 | A | C | T | G | A | C |
| SNPO157_20 | A | T | T | G | G | C |
| SNPO157_21 | C | T | T | G | G | C |
| SNPO157_22 | A | T | T | G | G | C |
| SNPO157_23 | A | T | T | G | G | C |
| SNPO157_24 | A | C | T | G | A | C |
| SNPO157_25 | A | T | T | G | G | C |
| SNPO157_26 | A | C | T | G | A | C |
| SNPO157_27 | A | T | T | G | G | C |
| SNPO157_28 | A | T | T | G | G | C |
| SNPO157_29 | A | C | T | G | A | C |
| SNPO157_30 | A | T | T | G | G | C |
| SNPO157_31 | A | T | T | G | G | C |
| SNPO157_32 | A | T | T | G | G | C |
| SNPO157_33 | A | T | T | G | G | C |
| SNPO157_34 | C | T | T | G | G | C |
| SNPO157_35 | C | T | T | G | G | C |
| SNPO157_36 | A | T | T | G | G | C |
| SNPO157_37 | C | T | T | G | G | C |
| SNPO157_38 | A | T | T | G | G | C |
| SNPO157_39 | C | T | T | G | G | C |

| | Locus tag (ORF designation) SNP position within ORF | | | | | |
|---|---|---|---|---|---|---|
| Strain ID | ECs3225.SNP-1098 | ECs3225.SNP-904 | ECs3231.SNP-1105 | ECs3246.SNP-1113 | ECs3246.SNP-743 | ECs3300.SNP-458 |
| 493/89 | T | G | C | C | G | G |
| 86-24 | C | G | C | C | A | G |
| 87-14 | C | G | C | C | A | G |
| CB9615 | T | G | T | T | G | G |
| EC4115 | C | G | C | C | A | A |
| EDL933 | C | G | C | C | A | G |
| LSU-61 | T | G | C | C | G | G |
| Sakai | C | G | C | C | A | G |
| TB182A | T | G | T | T | G | G |
| TW14359 | C | G | C | C | A | G |
| TW14588 | C | G | C | C | A | G |
| SNPO157_01 | C | G | C | C | A | G |
| SNPO157_02 | C | G | C | C | A | G |
| SNPO157_03 | T | G | C | C | G | G |
| SNPO157_04 | C | G | C | C | A | G |
| SNPO157_05 | C | G | C | C | A | G |
| SNPO157_06 | C | G | C | C | A | G |
| SNPO157_07 | C | G | C | C | A | G |
| SNPO157_08 | C | G | C | C | A | G |
| SNPO157_09 | T | G | C | C | G | G |
| SNPO157_10 | C | G | C | C | A | G |
| SNPO157_11 | T | G | C | C | G | G |
| SNPO157_12 | T | G | C | C | G | G |
| SNPO157_13 | C | G | C | C | A | G |
| SNPO157_14 | C | G | C | C | A | G |
| SNPO157_15 | C | G | C | C | A | G |
| SNPO157_16 | C | G | C | C | A | G |
| SNPO157_17 | C | G | C | C | A | G |
| SNPO157_18 | C | G | C | C | A | G |
| SNPO157_19 | C | G | C | C | A | G |
| SNPO157_20 | C | G | C | C | A | G |
| SNPO157_21 | T | G | C | C | G | G |
| SNPO157_22 | C | G | C | C | A | G |
| SNPO157_23 | C | G | C | C | A | G |
| SNPO157_24 | C | G | C | C | A | G |

| Strain ID | Locus tag (ORF designation) SNP position within ORF | | | | | |
|---|---|---|---|---|---|---|
| | ECs3225.SNP-1098 | ECs3225.SNP-904 | ECs3231.SNP-1105 | ECs3246.SNP-1113 | ECs3246.SNP-743 | ECs3300.SNP-458 |
| SNPO157_25 | C | G | C | C | A | G |
| SNPO157_26 | C | G | C | C | A | G |
| SNPO157_27 | C | G | C | C | A | G |
| SNPO157_28 | C | G | C | C | A | G |
| SNPO157_29 | C | G | C | C | A | G |
| SNPO157_30 | C | G | C | C | A | G |
| SNPO157_31 | C | G | C | C | A | G |
| SNPO157_32 | C | G | C | C | A | G |
| SNPO157_33 | C | G | C | C | A | G |
| SNPO157_34 | T | G | C | C | G | G |
| SNPO157_35 | T | G | C | C | G | G |
| SNPO157_36 | C | A | C | C | A | G |
| SNPO157_37 | T | G | C | C | G | G |
| SNPO157_38 | C | G | C | C | A | G |
| SNPO157_39 | T | G | C | C | G | G |

| Strain ID | Locus tag (ORF designation) SNP position within ORF | | | | | |
|---|---|---|---|---|---|---|
| | ECs3300.SNP-855 | ECs3377.SNP-522 | ECs3377.SNP-226 | ECs3377.SNP-127 | ECs3464.SNP-297 | ECs3464.SNP-227 |
| 493/89 | T | A | T | C | T | C |
| 86-24 | T | A | T | C | G | C |
| 87-14 | T | A | T | C | G | C |
| CB9615 | T | A | G | C | G | C |
| EC4115 | T | A | T | C | G | C |
| EDL933 | T | A | T | C | G | C |
| LSU-61 | T | A | T | T | G | C |
| Sakai | T | A | T | C | G | C |
| TB182A | A | A | G | C | G | C |
| TW14359 | T | A | T | C | G | C |
| TW14588 | T | A | T | C | G | C |
| SNPO157_01 | T | A | T | C | G | C |
| SNPO157_02 | T | A | T | C | G | C |
| SNPO157_03 | T | A | T | C | T | C |
| SNPO157_04 | T | A | T | C | G | C |
| SNPO157_05 | T | A | T | C | G | C |
| SNPO157_06 | T | A | T | C | G | C |
| SNPO157_07 | T | A | T | C | G | C |
| SNPO157_08 | T | C | T | C | G | C |
| SNPO157_09 | T | A | T | C | T | C |
| SNPO157_10 | T | A | T | C | G | C |
| SNPO157_11 | T | A | T | C | T | C |
| SNPO157_12 | T | A | T | C | T | C |
| SNPO157_13 | T | A | T | C | G | C |
| SNPO157_14 | T | A | T | C | G | C |
| SNPO157_15 | T | A | T | C | G | C |
| SNPO157_16 | T | A | T | C | G | C |
| SNPO157_17 | T | A | T | C | G | C |
| SNPO157_18 | T | A | T | C | G | C |
| SNPO157_19 | T | A | T | C | G | C |
| SNPO157_20 | T | A | T | C | G | C |
| SNPO157_21 | T | A | T | C | T | C |
| SNPO157_22 | T | A | T | C | G | C |
| SNPO157_23 | T | A | T | C | G | C |
| SNPO157_24 | T | A | T | C | G | C |
| SNPO157_25 | T | A | T | C | G | C |
| SNPO157_26 | T | A | T | C | G | C |
| SNPO157_27 | T | A | T | C | G | C |
| SNPO157_28 | T | A | T | C | G | C |
| SNPO157_29 | T | A | T | C | G | C |
| SNPO157_30 | T | A | T | C | G | C |
| SNPO157_31 | T | A | T | C | G | C |
| SNPO157_32 | T | A | T | C | G | A |
| SNPO157_33 | T | A | T | C | G | C |
| SNPO157_34 | T | A | T | C | T | C |
| SNPO157_35 | T | A | T | C | T | C |

| Strain ID | Locus tag (ORF designation) SNP position within ORF | | | | | |
|---|---|---|---|---|---|---|
| | ECs3300.SNP-855 | ECs3377.SNP-522 | ECs3377.SNP-226 | ECs3377.SNP-127 | ECs3464.SNP-297 | ECs3464.SNP-227 |
| SNPO157_36 | T | A | T | C | G | C |
| SNPO157_37 | T | A | T | C | T | C |
| SNPO157_38 | T | A | T | C | G | C |
| SNPO157_39 | T | A | T | C | T | C |

| Strain ID | Locus tag (ORF designation) SNP position within ORF | | | | | |
|---|---|---|---|---|---|---|
| | ECs3564.SNP-786 | ECs3676.SNP-147 | ECs3746.SNP-984 | ECs3746.SNP-1136 | ECs3773.SNP-1100 | ECs3810.SNP-429 |
| 493/89 | C | C | T | A | A | A |
| 86-24 | C | C | C | A | A | G |
| 87-14 | A | C | C | A | A | G |
| CB9615 | C | T | C | A | C | G |
| EC4115 | A | C | C | A | A | G |
| EDL933 | C | C | C | A | A | G |
| LSU-61 | C | C | C | A | A | G |
| Sakai | C | C | C | A | A | G |
| TB182A | C | T | C | A | C | G |
| TW14359 | A | C | C | A | A | G |
| TW14588 | C | C | C | A | A | G |
| SNPO157_01 | A | C | C | A | A | G |
| SNPO157_02 | A | C | C | A | A | G |
| SNPO157_03 | C | C | T | A | A | A |
| SNPO157_04 | A | C | C | A | A | G |
| SNPO157_05 | C | C | C | A | A | G |
| SNPO157_06 | C | C | C | A | A | G |
| SNPO157_07 | A | C | C | A | A | G |
| SNPO157_08 | A | C | C | A | A | G |
| SNPO157_09 | C | C | T | A | A | A |
| SNPO157_10 | A | C | C | A | A | G |
| SNPO157_11 | C | C | T | A | A | A |
| SNPO157_12 | C | C | T | A | A | A |
| SNPO157_13 | A | C | C | A | A | G |
| SNPO157_14 | A | C | C | A | A | G |
| SNPO157_15 | A | C | C | A | A | G |
| SNPO157_16 | A | C | C | A | A | G |
| SNPO157_17 | A | C | C | A | A | G |
| SNPO157_18 | C | C | C | A | A | G |
| SNPO157_19 | A | C | C | A | A | G |
| SNPO157_20 | C | C | C | A | A | G |
| SNPO157_21 | C | C | T | A | A | A |
| SNPO157_22 | C | C | C | A | A | G |
| SNPO157_23 | C | C | C | A | A | G |
| SNPO157_24 | A | C | C | A | A | G |
| SNPO157_25 | C | C | C | C | A | G |
| SNPO157_26 | A | C | C | A | A | G |
| SNPO157_27 | C | C | C | A | A | G |
| SNPO157_28 | C | C | C | A | A | G |
| SNPO157_29 | A | C | C | A | A | G |
| SNPO157_30 | C | C | C | A | A | G |
| SNPO157_31 | C | C | C | A | A | G |
| SNPO157_32 | C | C | C | C | A | G |
| SNPO157_33 | C | C | C | A | A | G |
| SNPO157_34 | C | C | T | A | A | A |
| SNPO157_35 | C | C | T | A | A | A |
| SNPO157_36 | C | C | C | A | A | G |
| SNPO157_37 | C | C | T | A | A | A |
| SNPO157_38 | C | C | C | A | A | G |
| SNPO157_39 | C | C | T | A | A | A |

| Strain ID | Locus tag (ORF designation) SNP position within ORF | | | | | |
|---|---|---|---|---|---|---|
| | ECs3833.SNP-365 | ECs3839.SNP-793 | ECs3994.SNP-1663 | ECs4047.SNP-272 | ECs4109.SNP-873 | ECs4109.SNP-812 |
| 493/89 | T | G | C | G | C | T |
| 86-24 | T | G | T | G | C | C |

| Strain ID | Locus tag (ORF designation) SNP position within ORF | | | | | |
|---|---|---|---|---|---|---|
| | ECs3833.SNP-365 | ECs3839.SNP-793 | ECs3994.SNP-1663 | ECs4047.SNP-272 | ECs4109.SNP-873 | ECs4109.SNP-812 |
| 87-14 | T | G | T | G | C | C |
| CB9615 | T | A | C | G | A | C |
| EC4115 | T | G | T | A | C | C |
| EDL933 | T | G | T | G | C | C |
| LSU-61 | T | G | C | G | C | C |
| Sakai | T | G | T | G | C | C |
| TB182A | C | A | C | G | A | C |
| TW14359 | T | G | T | A | C | C |
| TW14588 | T | G | T | G | C | C |
| SNPO157_01 | T | G | T | G | C | C |
| SNPO157_02 | T | G | T | G | C | C |
| SNPO157_03 | T | G | C | G | C | T |
| SNPO157_04 | T | G | T | G | C | C |
| SNPO157_05 | T | G | T | G | C | C |
| SNPO157_06 | T | G | T | G | C | C |
| SNPO157_07 | T | G | T | G | C | C |
| SNPO157_08 | T | G | T | G | C | C |
| SNPO157_09 | T | G | C | G | C | T |
| SNPO157_10 | T | G | T | G | C | C |
| SNPO157_11 | T | G | C | G | C | T |
| SNPO157_12 | T | G | C | G | C | T |
| SNPO157_13 | T | G | T | G | C | C |
| SNPO157_14 | T | G | T | G | C | C |
| SNPO157_15 | T | G | T | G | C | C |
| SNPO157_16 | T | G | T | G | C | C |
| SNPO157_17 | T | G | T | G | C | C |
| SNPO157_18 | T | G | T | G | C | C |
| SNPO157_19 | T | G | T | G | C | C |
| SNPO157_20 | T | G | T | G | C | C |
| SNPO157_21 | T | G | C | G | C | T |
| SNPO157_22 | T | G | T | G | C | C |
| SNPO157_23 | T | G | T | G | C | C |
| SNPO157_24 | T | G | T | G | C | C |
| SNPO157_25 | T | G | T | G | C | C |
| SNPO157_26 | T | G | T | G | C | C |
| SNPO157_27 | T | G | T | G | C | C |
| SNPO157_28 | T | G | T | G | C | C |
| SNPO157_29 | T | G | T | G | C | C |
| SNPO157_30 | T | G | T | G | C | C |
| SNPO157_31 | T | G | T | G | C | C |
| SNPO157_32 | T | G | T | G | C | C |
| SNPO157_33 | T | G | T | G | C | C |
| SNPO157_34 | T | G | C | G | C | T |
| SNPO157_35 | T | G | C | G | C | T |
| SNPO157_36 | T | G | T | G | C | C |
| SNPO157_37 | T | G | C | G | C | T |
| SNPO157_38 | T | G | T | G | C | C |
| SNPO157_39 | T | G | C | G | C | T |

| Strain ID | Locus tag (ORF designation) SNP position within ORF | | | | | | |
|---|---|---|---|---|---|---|---|
| | ECs4109.SNP-312 | ECs4130.SNP-624 | ECs4130.SNP-867 | ECs4204.SNP-380 | ECs4204.SNP-618 | ECs4226.SNP-738 | ECs4259.SNP-1680 |
| 493/89 | T | C | G | A | A | T | T |
| 86-24 | T | C | A | A | G | C | T |
| 87-14 | T | C | A | A | G | C | T |
| CB9615 | T | C | G | A | G | C | C |
| EC4115 | T | C | A | A | G | C | T |
| EDL933 | T | T | A | A | G | C | T |
| LSU-61 | T | C | G | C | G | C | T |
| Sakai | T | T | A | A | G | C | T |
| TB182A | T | C | G | A | G | C | C |
| TW14359 | T | C | A | A | G | C | T |
| TW14588 | T | T | A | A | G | C | T |
| SNPO157_01 | T | C | A | A | G | C | T |
| SNPO157_02 | T | C | A | A | G | C | T |

| | Locus tag (ORF designation) SNP position within ORF | | | | | | |
|---|---|---|---|---|---|---|---|
| Strain ID | ECs4109.SNP -312 | ECs4130.SNP -624 | ECs4130.SNP -867 | ECs4204.SNP -380 | ECs4204.SNP -618 | ECs4226.SNP -738 | ECs4259.SNP -1680 |
| SNPO157_03 | T | C | G | A | A | T | T |
| SNPO157_04 | T | C | A | A | G | C | T |
| SNPO157_05 | T | C | A | A | G | C | T |
| SNPO157_06 | T | C | A | A | G | C | T |
| SNPO157_07 | T | C | A | A | G | C | T |
| SNPO157_08 | T | C | A | A | G | C | T |
| SNPO157_09 | T | C | G | A | A | T | T |
| SNPO157_10 | T | C | A | A | G | C | T |
| SNPO157_11 | T | C | G | A | A | T | T |
| SNPO157_12 | T | C | G | A | A | T | T |
| SNPO157_13 | T | C | A | A | G | C | T |
| SNPO157_14 | T | C | A | A | G | C | T |
| SNPO157_15 | T | C | A | A | G | C | T |
| SNPO157_16 | T | C | A | A | G | C | T |
| SNPO157_17 | T | C | A | A | G | C | T |
| SNPO157_18 | T | C | A | A | G | C | T |
| SNPO157_19 | T | C | A | A | G | C | T |
| SNPO157_20 | T | T | A | A | G | C | T |
| SNPO157_21 | T | C | G | A | A | T | T |
| SNPO157_22 | T | C | A | A | G | C | T |
| SNPO157_23 | T | T | A | A | G | C | T |
| SNPO157_24 | T | C | A | A | G | C | T |
| SNPO157_25 | T | C | A | A | G | C | T |
| SNPO157_26 | T | C | A | A | G | C | T |
| SNPO157_27 | T | C | A | A | G | C | T |
| SNPO157_28 | T | C | A | A | G | C | T |
| SNPO157_29 | T | C | A | A | G | C | T |
| SNPO157_30 | T | C | A | A | G | C | T |
| SNPO157_31 | T | C | A | A | G | C | T |
| SNPO157_32 | T | C | A | A | G | C | T |
| SNPO157_33 | T | C | A | A | G | C | T |
| SNPO157_34 | T | C | G | A | A | T | T |
| SNPO157_35 | T | C | G | A | A | T | T |
| SNPO157_36 | T | C | G | A | G | C | T |
| SNPO157_37 | T | C | G | A | A | T | T |
| SNPO157_38 | T | C | A | A | G | C | T |
| SNPO157_39 | C | C | G | A | A | T | T |

| | Locus tag (ORF designation) SNP position within ORF | | | | | | |
|---|---|---|---|---|---|---|---|
| Strain ID | ECs4265.SNP -609 | ECs4359.SNP -1726 | ECs4359.SNP -1519 | ECs4364.SNP -1079 | ECs4411.SNP -590 | ECs4411.SNP -366 | ECs4411.SNP -324 |
| 493/89 | G | G | G | G | G | G | C |
| 86-24 | A | G | G | G | G | A | C |
| 87-14 | A | G | G | G | G | A | C |
| CB9615 | G | A | G | A | G | G | C |
| EC4115 | A | G | A | G | G | A | C |
| EDL933 | A | G | G | G | G | A | C |
| LSU-61 | G | G | G | G | G | G | C |
| Sakai | A | G | G | G | G | A | C |
| TB182A | G | A | G | A | G | G | C |
| TW14359 | A | G | A | G | G | A | C |
| TW14588 | A | G | G | G | G | A | C |
| SNPO157_01 | A | G | G | G | G | A | C |
| SNPO157_02 | A | G | A | G | G | A | C |
| SNPO157_03 | G | G | G | G | G | G | C |
| SNPO157_04 | A | G | G | G | G | A | C |
| SNPO157_05 | A | G | G | G | G | A | C |
| SNPO157_06 | A | G | G | G | G | A | C |
| SNPO157_07 | A | G | A | G | G | A | C |
| SNPO157_08 | A | G | G | G | G | A | C |
| SNPO157_09 | G | G | G | G | G | G | C |
| SNPO157_10 | A | G | G | G | G | A | C |
| SNPO157_11 | G | G | G | G | G | G | C |
| SNPO157_12 | G | G | G | G | G | G | C |
| SNPO157_13 | A | G | A | G | G | A | C |

| Strain ID | Locus tag (ORF designation) SNP position within ORF | | | | | | |
|---|---|---|---|---|---|---|---|
| | ECs4265.SNP -609 | ECs4359.SNP -1726 | ECs4359.SNP -1519 | ECs4364.SNP -1079 | ECs4411.SNP -590 | ECs4411.SNP -366 | ECs4411.SNP -324 |
| SNPO157_14 | A | G | G | G | G | A | C |
| SNPO157_15 | A | G | G | G | G | A | C |
| SNPO157_16 | A | G | G | G | G | A | C |
| SNPO157_17 | A | G | G | G | G | A | C |
| SNPO157_18 | A | G | G | G | G | A | C |
| SNPO157_19 | A | G | G | G | G | A | C |
| SNPO157_20 | A | G | G | G | G | A | C |
| SNPO157_21 | G | G | G | G | G | G | C |
| SNPO157_22 | A | G | G | G | G | A | C |
| SNPO157_23 | A | G | G | G | G | A | C |
| SNPO157_24 | A | G | G | G | G | A | C |
| SNPO157_25 | A | G | G | G | G | A | C |
| SNPO157_26 | A | G | G | G | G | A | C |
| SNPO157_27 | A | G | G | G | G | A | C |
| SNPO157_28 | A | G | G | G | A | A | C |
| SNPO157_29 | A | G | G | G | G | A | C |
| SNPO157_30 | A | G | G | G | G | A | C |
| SNPO157_31 | A | G | G | G | A | A | C |
| SNPO157_32 | A | G | G | G | G | A | C |
| SNPO157_33 | A | G | G | G | A | A | C |
| SNPO157_34 | G | G | G | G | G | G | C |
| SNPO157_35 | G | G | G | G | G | G | C |
| SNPO157_36 | A | G | G | G | G | A | T |
| SNPO157_37 | G | G | G | G | G | G | C |
| SNPO157_38 | A | G | G | G | G | A | C |
| SNPO157_39 | G | G | G | G | G | G | C |

| Strain ID | Locus tag (ORF designation) SNP position within ORF | | | | | | |
|---|---|---|---|---|---|---|---|
| | ECs4418.SNP-467 | ECs4418.SNP-582 | ECs4424.SNP-192 | ECs4454.SNP-165 | ECs4454.SNP-402 | ECs4493.SNP-509 | ECs4514.SNP-684 |
| 493/89 | G | T | T | A | C | A | C |
| 86-24 | G | G | T | G | C | A | C |
| 87-14 | G | G | T | G | C | A | A |
| CB9615 | G | G | C | A | C | A | C |
| EC4115 | G | G | T | G | C | A | C |
| EDL933 | G | G | T | G | C | A | C |
| LSU-61 | G | G | T | A | C | A | C |
| Sakai | G | G | T | G | C | A | C |
| TB182A | G | G | C | A | A | A | C |
| TW14359 | G | G | T | G | C | A | C |
| TW14588 | G | G | T | G | C | A | C |
| SNPO157_01 | G | G | T | G | C | A | A |
| SNPO157_02 | G | G | T | G | C | A | C |
| SNPO157_03 | G | T | T | A | C | A | C |
| SNPO157_04 | G | G | T | G | C | A | A |
| SNPO157_05 | G | G | T | G | C | A | C |
| SNPO157_06 | G | G | T | G | C | A | C |
| SNPO157_07 | G | G | T | G | C | A | C |
| SNPO157_08 | G | G | T | G | C | A | C |
| SNPO157_09 | A | T | T | A | C | A | C |
| SNPO157_10 | G | G | T | G | C | A | A |
| SNPO157_11 | G | T | T | A | C | A | C |
| SNPO157_12 | G | T | T | A | C | A | C |
| SNPO157_13 | G | G | T | G | C | A | C |
| SNPO157_14 | G | G | T | G | C | A | A |
| SNPO157_15 | G | G | T | G | C | A | A |
| SNPO157_16 | G | G | T | G | C | A | A |
| SNPO157_17 | G | G | T | G | C | A | A |
| SNPO157_18 | G | G | T | G | C | A | C |
| SNPO157_19 | G | G | T | G | C | A | A |
| SNPO157_20 | G | G | T | G | C | A | C |
| SNPO157_21 | G | T | T | A | C | A | C |
| SNPO157_22 | G | G | T | G | C | A | C |
| SNPO157_23 | G | G | T | G | C | A | C |
| SNPO157_24 | G | G | T | G | C | A | A |

| Strain ID | Locus tag (ORF designation) SNP position within ORF | | | | | | |
|---|---|---|---|---|---|---|---|
| | ECs4418.SNP-467 | ECs4418.SNP-582 | ECs4424.SNP-192 | ECs4454.SNP-165 | ECs4454.SNP-402 | ECs4493.SNP-509 | ECs4514.SNP-684 |
| SNPO157_25 | G | G | T | G | C | A | C |
| SNPO157_26 | G | G | T | G | C | C | A |
| SNPO157_27 | G | G | T | G | C | A | C |
| SNPO157_28 | G | G | T | G | C | A | C |
| SNPO157_29 | G | G | T | G | C | A | A |
| SNPO157_30 | G | G | T | G | C | A | C |
| SNPO157_31 | G | G | T | G | C | A | C |
| SNPO157_32 | G | G | T | G | C | A | C |
| SNPO157_33 | G | G | T | G | C | A | C |
| SNPO157_34 | G | T | T | A | C | A | C |
| SNPO157_35 | G | T | T | A | C | A | C |
| SNPO157_36 | G | G | T | G | C | A | C |
| SNPO157_37 | G | T | T | A | C | A | C |
| SNPO157_38 | G | G | T | G | C | A | C |
| SNPO157_39 | G | T | T | A | C | A | C |

| Strain ID | Locus tag (ORF designation) SNP position within ORF | | | | | | |
|---|---|---|---|---|---|---|---|
| | ECs4601.SNP-763 | ECs4689.SNP-683 | ECs4711.SNP-57 | ECs4841.SNP-717 | ECs4861.SNP-471 | ECs4872.SNP-261 | ECs4920.SNP-568 |
| 493/89 | T | T | G | C | C | G | G |
| 86-24 | C | T | G | C | C | G | G |
| 87-14 | C | G | G | C | C | G | G |
| CB9615 | C | T | A | C | C | G | A |
| EC4115 | C | G | G | C | C | G | G |
| EDL933 | C | T | G | C | C | G | G |
| LSU-61 | C | T | G | C | C | G | G |
| Sakai | C | T | G | C | C | G | G |
| TB182A | C | T | A | C | C | G | A |
| TW14359 | C | G | G | C | C | G | G |
| TW14588 | C | T | G | C | C | G | G |
| SNPO157_01 | C | G | G | C | C | G | G |
| SNPO157_02 | C | G | G | C | C | G | G |
| SNPO157_03 | T | T | G | C | C | G | G |
| SNPO157_04 | C | G | G | C | C | G | G |
| SNPO157_05 | C | T | G | C | C | G | G |
| SNPO157_06 | C | T | G | C | C | G | G |
| SNPO157_07 | C | G | G | C | C | G | G |
| SNPO157_08 | C | G | G | C | C | G | G |
| SNPO157_09 | T | T | G | C | C | G | G |
| SNPO157_10 | C | G | G | C | C | G | G |
| SNPO157_11 | T | T | G | C | C | G | G |
| SNPO157_12 | T | T | G | C | C | G | G |
| SNPO157_13 | C | G | G | C | C | G | G |
| SNPO157_14 | C | G | G | C | C | G | G |
| SNPO157_15 | C | G | G | C | C | G | G |
| SNPO157_16 | C | G | G | C | C | G | G |
| SNPO157_17 | C | G | G | C | C | G | G |
| SNPO157_18 | C | T | G | C | C | G | G |
| SNPO157_19 | C | G | G | C | C | G | G |
| SNPO157_20 | C | T | G | C | C | G | G |
| SNPO157_21 | T | T | G | C | C | G | G |
| SNPO157_22 | C | T | G | C | C | G | G |
| SNPO157_23 | C | T | G | T | C | G | G |
| SNPO157_24 | C | G | G | C | T | G | G |
| SNPO157_25 | C | T | G | C | C | G | G |
| SNPO157_26 | C | G | G | C | C | G | G |
| SNPO157_27 | C | T | G | C | C | G | G |
| SNPO157_28 | C | T | G | C | C | A | G |
| SNPO157_29 | C | G | G | C | C | G | G |
| SNPO157_30 | C | T | G | C | C | G | G |
| SNPO157_31 | C | T | G | C | C | A | G |
| SNPO157_32 | C | T | G | C | C | G | G |
| SNPO157_33 | C | T | G | C | C | A | G |
| SNPO157_34 | T | T | G | C | C | G | G |
| SNPO157_35 | T | T | G | C | C | G | G |

| | Locus tag (ORF designation) SNP position within ORF | | | | | | |
|---|---|---|---|---|---|---|---|
| Strain ID | ECs4601.SNP -763 | ECs4689.SNP -683 | ECs4711.SNP -57 | ECs4841.SNP -717 | ECs4861.SNP -471 | ECs4872.SNP -261 | ECs4920.SNP -568 |
| SNPO157_36 | C | T | G | C | C | G | G |
| SNPO157_37 | T | T | G | C | C | G | G |
| SNPO157_38 | C | T | G | C | C | G | G |
| SNPO157_39 | T | T | G | C | C | G | G |

| | Locus tag (ORF designation) SNP position within ORF | | | |
|---|---|---|---|---|
| Strain ID | ECs5210.SNP-308 | ECs5210.SNP-366 | ECs5222.SNP-344 | ECs5222.SNP-70 |
| 493/89 | G | T | T | C |
| 86-24 | G | T | A | C |
| 87-14 | G | T | A | T |
| CB9615 | G | C | A | C |
| EC4115 | G | T | A | C |
| EDL933 | G | T | A | C |
| LSU-61 | G | T | A | C |
| Sakai | A | T | A | C |
| TB182A | G | C | A | C |
| TW14359 | G | T | A | C |
| TW14588 | A | T | A | C |
| SNPO157_01 | G | T | A | C |
| SNPO157_02 | G | T | A | C |
| SNPO157_03 | G | T | T | C |
| SNPO157_04 | G | T | A | C |
| SNPO157_05 | G | T | A | C |
| SNPO157_06 | G | T | A | C |
| SNPO157_07 | G | T | A | C |
| SNPO157_08 | G | T | A | C |
| SNPO157_09 | G | T | T | C |
| SNPO157_10 | G | T | A | C |
| SNPO157_11 | G | T | T | C |
| SNPO157_12 | G | T | T | C |
| SNPO157_13 | G | T | A | C |
| SNPO157_14 | G | T | A | C |
| SNPO157_15 | G | T | A | C |
| SNPO157_16 | G | T | A | C |
| SNPO157_17 | G | T | A | C |
| SNPO157_18 | G | T | A | C |
| SNPO157_19 | G | T | A | C |
| SNPO157_20 | A | T | A | C |
| SNPO157_21 | G | T | T | C |
| SNPO157_22 | G | T | A | C |
| SNPO157_23 | G | T | A | C |
| SNPO157_24 | G | T | A | C |
| SNPO157_25 | G | T | A | C |
| SNPO157_26 | G | T | A | C |
| SNPO157_27 | G | T | A | C |
| SNPO157_28 | G | T | A | C |
| SNPO157_29 | G | T | A | C |
| SNPO157_30 | G | T | A | C |
| SNPO157_31 | G | T | A | C |
| SNPO157_32 | G | T | A | C |
| SNPO157_33 | G | T | A | C |
| SNPO157_34 | G | T | T | C |
| SNPO157_35 | G | T | T | C |
| SNPO157_36 | G | T | A | C |
| SNPO157_37 | G | T | T | C |
| SNPO157_38 | G | T | A | C |
| SNPO157_39 | G | T | T | C |

Technical Appendix Table 3. List of all 111 SNPs discovered in this study

| ORF designation | ORF orientation within the Genome | SNP Position within the ORF | SNP Position within the Genome | Reference Nucleotide | Variant Nucleotide | Reference Codon | Variant Codon | Reference Amino acid | Variant Amino acid | Codon Effect* |
|---|---|---|---|---|---|---|---|---|---|---|
| ECs0076 | - | 1212 | 84260 | C | T | TTG | TTA | L | L | syn |
| ECs0076 | - | 1164 | 84308 | T | C | GCA | GCG | A | A | syn |
| ECs0093 | + | 687 | 103694 | C | T | GGC | GGT | G | G | syn |
| ECs0100 | + | 406 | 111567 | G | A | GTT | ATT | V | I | non-syn |
| ECs0204 | - | 573 | 233638 | C | T | CTG | CTA | L | L | syn |
| ECs0406 | + | 494 | 432014 | C | T | CCT | CTT | P | L | non-syn |
| ECs0444 | + | 260 | 472489 | C | T | CCA | CTA | P | L | non-syn |
| ECs0502 | + | 342 | 535275 | T | G | GGT | GGG | G | G | syn |
| ECs0523 | + | 1134 | 559120 | C | T | GTC | GTT | V | V | syn |
| ECs0552 | - | 186 | 609385 | C | T | ATG | ATA | M | I | non-syn |
| ECs0614 | + | 138 | 684625 | C | T | GGC | GGT | G | G | syn |
| ECs0614 | + | 283 | 684770 | T | A | TGG | AGG | W | R | non-syn |
| ECs0647 | + | 216 | 723657 | T | C | GAT | GAC | D | D | syn |
| ECs0647 | + | 306 | 723747 | C | T | GCC | GCT | A | A | syn |
| ECs0647 | + | 556 | 723997 | C | G | CAA | GAA | Q | E | non-syn |
| ECs0905 | - | 453 | 990246 | C | T | CCG | CCA | P | P | syn |
| ECs0905 | - | 217 | 990482 | G | C | CCG | GCG | P | A | non-syn |
| ECs0917 | + | 578 | 1004607 | C | T | CCG | CTG | P | L | non-syn |
| ECs0955 | - | 745 | 1038649 | C | A | GTG | TTG | V | L | non-syn |
| ECs0955 | - | 719 | 1038675 | C | T | CGC | CAC | R | H | non-syn |
| ECs0998 | + | 375 | 1098382 | C | T | CCC | CCT | P | P | syn |
| ECs1044 | - | 2080 | 1151284 | A | G | TTA | CTA | L | L | syn |
| ECs1044 | - | 1773 | 1151591 | C | T | GTG | GTA | V | V | syn |
| ECs1046 | + | 319 | 1154260 | C | T | CTG | TTG | L | L | syn |
| ECs1046 | + | 358 | 1154299 | C | A | CTG | ATG | L | M | non-syn |
| ECs1137 | - | 524 | 1222818 | A | G | ATG | ACG | M | T | non-syn |
| ECs1137 | - | 230 | 1223112 | C | T | AGT | AAT | S | N | non-syn |
| ECs1148 | - | 1014 | 1233518 | C | A | ATG | ATT | M | I | non-syn |
| ECs1148 | - | 940 | 1233592 | G | A | CGT | TGT | R | C | non-syn |
| ECs1729 | + | 786 | 1725872 | C | T | ACC | ACT | T | T | syn |
| ECs1741 | - | 2155 | 1740010 | G | A | CTG | TTG | L | L | syn |
| ECs1833 | - | 474 | 1818962 | C | T | CCG | CCA | P | P | syn |
| ECs1871 | - | 1109 | 1863935 | A | G | TTG | TCG | L | S | non-syn |
| ECs1871 | - | 978 | 1864066 | G | A | CGC | CGT | R | R | syn |
| ECs1878 | + | 829 | 1872868 | G | T | GGC | TGC | G | C | non-syn |
| ECs2022 | - | 793 | 2007430 | T | A | ACC | TCC | T | S | non-syn |
| ECs2045 | + | 677 | 2029256 | C | T | CCG | CTG | P | L | non-syn |
| ECs2087 | - | 253 | 2079029 | T | C | ATG | GTG | M | V | non-syn |
| ECs2320 | + | 486 | 2290138 | A | G | CTA | CTG | L | L | syn |
| ECs2320 | + | 719 | 2290371 | A | T | GAA | GTA | E | V | non-syn |
| ECs2435 | + | 123 | 2410770 | G | A | GTG | GTA | V | V | syn |
| ECs2435 | + | 458 | 2411105 | T | A | ATC | AAC | I | N | non-syn |
| ECs2454 | - | 1064 | 2430431 | G | A | TCT | TTT | S | F | non-syn |
| ECs2454 | - | 693 | 2430802 | A | G | CGT | CGC | R | R | syn |
| ECs2582 | - | 2038 | 2556900 | G | A | CGT | TGT | R | C | non-syn |
| ECs2583 | - | 73 | 2559990 | T | G | ATG | CTG | M | L | non-syn |
| ECs2589 | - | 804 | 2566177 | A | C | GTT | GTG | V | V | syn |
| ECs2589 | - | 771 | 2566210 | A | C | GCT | GCG | A | A | syn |
| ECs3043 | - | 721 | 2975941 | C | T | GTA | ATA | V | I | non-syn |
| ECs3059 | - | 1440 | 2994900 | A | C | CCT | CCG | P | P | syn |
| ECs3076 | + | 311 | 3015872 | T | C | CTT | CCT | L | P | non-syn |
| ECs3076 | + | 573 | 3016134 | T | G | ATT | ATG | I | M | non-syn |
| ECs3076 | + | 1413 | 3016974 | G | A | GCG | GCA | A | A | syn |
| ECs3076 | + | 1689 | 3017250 | G | A | CAG | CAA | Q | Q | syn |
| ECs3207 | - | 291 | 3167678 | C | A | CCG | CCT | P | P | syn |
| ECs3225 | - | 1098 | 3185787 | C | T | GCG | GCA | A | A | syn |
| ECs3225 | - | 904 | 3185981 | G | A | CGC | TGC | R | C | non-syn |
| ECs3231 | + | 1105 | 3194248 | C | T | CGC | TGC | R | C | non-syn |
| ECs3246 | - | 1113 | 3209201 | C | T | CCG | CCA | P | P | syn |
| ECs3246 | - | 743 | 3209571 | A | G | TTT | TCT | F | S | non-syn |
| ECs3300 | + | 458 | 3270415 | G | A | GGC | GAC | G | D | non-syn |
| ECs3300 | + | 855 | 3270812 | T | A | GGT | GGA | G | G | syn |
| ECs3377 | - | 522 | 3357424 | A | C | GAT | GAG | D | E | non-syn |
| ECs3377 | - | 226 | 3357720 | T | G | AAA | CAA | K | Q | non-syn |
| ECs3377 | - | 127 | 3357819 | C | T | GCA | ACA | A | T | non-syn |

| ORF designation | ORF orientation within the Genome | SNP Position within the ORF | SNP Position within the Genome | Reference Nucleotide | Variant Nucleotide | Reference Codon | Variant Codon | Reference Amino acid | Variant Amino acid | Codon Effect* |
|---|---|---|---|---|---|---|---|---|---|---|
| ECs3464 | - | 297 | 3460973 | G | T | CCC | CCA | P | P | syn |
| ECs3464 | - | 227 | 3461043 | C | A | CGA | CTA | R | L | non-syn |
| ECs3564 | + | 786 | 3554521 | C | A | ACC | ACA | T | T | syn |
| ECs3676 | - | 147 | 3674715 | C | T | GGG | GGA | G | G | syn |
| ECs3746 | + | 984 | 3749342 | C | T | GGC | GGT | G | G | syn |
| ECs3746 | + | 1136 | 3749494 | A | C | CAA | CCA | Q | P | non-syn |
| ECs3773 | + | 1100 | 3782791 | A | C | GAG | GCG | E | A | non-syn |
| ECs3810 | - | 429 | 3821818 | G | A | GAC | GAT | D | D | syn |
| ECs3833 | - | 365 | 3841958 | T | C | GAC | GGC | D | G | non-syn |
| ECs3839 | + | 793 | 3846816 | G | A | GCC | ACC | A | T | non-syn |
| ECs3994 | - | 1663 | 4000385 | T | C | AAA | GAA | K | E | non-syn |
| ECs4047 | - | 272 | 4055003 | G | A | GCC | GTC | A | V | non-syn |
| ECs4109 | - | 873 | 4118633 | C | A | GCG | GCT | A | A | syn |
| ECs4109 | - | 812 | 4118694 | C | T | GGT | GAT | G | D | non-syn |
| ECs4109 | - | 312 | 4119194 | T | C | CAA | CAG | Q | Q | syn |
| ECs4130 | + | 624 | 4143190 | T | C | GGT | GGC | G | G | syn |
| ECs4130 | + | 867 | 4143433 | A | G | CTA | CTG | L | L | syn |
| ECs4204 | + | 380 | 4203487 | A | C | GAA | GCA | E | A | non-syn |
| ECs4204 | + | 618 | 4203725 | G | A | CTG | CTA | L | L | syn |
| ECs4226 | - | 738 | 4225097 | C | T | CTG | CTA | L | L | syn |
| ECs4259 | - | 1680 | 4262902 | T | C | CCA | CCG | P | P | syn |
| ECs4265 | + | 609 | 4271823 | A | G | GTA | GTG | V | V | syn |
| ECs4359 | - | 1726 | 4362239 | G | A | CGC | TGC | R | C | non-syn |
| ECs4359 | - | 1519 | 4362446 | G | A | CCT | TCT | P | S | non-syn |
| ECs4364 | - | 1079 | 4370817 | G | A | CCT | CTT | P | L | non-syn |
| ECs4411 | - | 590 | 4431800 | G | A | CCG | CTG | P | L | non-syn |
| ECs4411 | - | 366 | 4432024 | A | G | GCT | GCC | A | A | syn |
| ECs4411 | - | 324 | 4432066 | C | T | GTG | GTA | V | V | syn |
| ECs4418 | + | 467 | 4440826 | G | A | GGA | GAA | G | E | non-syn |
| ECs4418 | + | 582 | 4440941 | G | T | ATG | ATT | M | I | non-syn |
| ECs4424 | - | 192 | 4449503 | T | C | CTA | CTG | L | L | syn |
| ECs4454 | + | 165 | 4483743 | G | A | CAG | CAA | Q | Q | syn |
| ECs4454 | + | 402 | 4483980 | C | A | AGC | AGA | S | R | non-syn |
| ECs4493 | - | 509 | 4536406 | A | C | GTT | GGT | V | G | non-syn |
| ECs4514 | + | 684 | 4557273 | C | A | GGC | GGA | G | G | syn |
| ECs4601 | - | 763 | 4630237 | C | T | GGC | AGC | G | S | non-syn |
| ECs4689 | + | 683 | 4725442 | T | G | GTC | GGC | V | G | non-syn |
| ECs4711 | + | 57 | 4754139 | G | A | CTG | CTA | L | L | syn |
| ECs4841 | + | 717 | 4908228 | C | T | ACC | ACT | T | T | syn |
| ECs4861 | - | 471 | 4925478 | C | T | CCG | CCA | P | P | syn |
| ECs4872 | + | 261 | 4943251 | G | A | GCG | GCA | A | A | syn |
| ECs4920 | + | 568 | 5007108 | G | A | GCT | ACT | A | T | non-syn |
| ECs5210 | + | 308 | 5311572 | A | G | CAC | CGC | H | R | non-syn |
| ECs5210 | + | 366 | 5311630 | T | C | ACT | ACC | T | T | syn |
| ECs5222 | - | 344 | 5327590 | A | T | CTG | CAG | L | Q | non-syn |
| ECs5222 | - | 70 | 5327864 | C | T | GTG | ATG | V | M | non-syn |

*syn, synonymous; non-syn, non-synonymous
